# Getting the dose right using physiologically-based pharmacokinetic modeling: dexamethasone to prevent post-extubation stridor in children as proof of concept

**DOI:** 10.3389/fped.2024.1416440

**Published:** 2024-07-05

**Authors:** Joyce E. M. van der Heijden, Marika de Hoop-Sommen, Noa Hoevenaars, Jolien J. M. Freriksen, Koen Joosten, Rick Greupink, Saskia N. de Wildt

**Affiliations:** ^1^ Division of Pharmacology and Toxicology, Department of Pharmacy, Radboud University Medical Center, Nijmegen, Netherlands; ^2^ Department of Neonatal and Pediatric Intensive Care, Division of Pediatric Intensive Care, Erasmus MC-Sophia Children’s Hospital, Rotterdam, Netherlands

**Keywords:** dexamethasone, modeling & simulation, pediatrics, pharmacokinetics, physiologically-based pharmacokinetic modeling, special populations

## Abstract

**Introduction:**

Critically ill patients show large variability in drug disposition due to e.g., age, size, disease and treatment modalities. Physiologically-based pharmacokinetic (PBPK) models can be used to design individualized dosing regimens taking this into account. Dexamethasone, prescribed for the prevention post-extubation stridor (PES), is metabolized by the drug metabolizing enzyme CYP3A. As CYP3A4 undergoes major changes during childhood, we aimed to develop age-appropriate dosing recommendations for children of dexamethasone for PES, as proof of concept for PBPK modeling to individualize dosing for critically ill patients.

**Methods:**

All simulations were conducted in Simcyp™ v21 (a population-based PBPK modeling platform), using an available dexamethasone compound model and pediatric population model in which CYP3A4 ontogeny is incorporated. Published pharmacokinetic (PK) data was used for model verification. Evidence for the dose to prevent post-extubation stridor was strongest for 2–6 year old children, hence simulated drug concentrations resulting from this dose from this age group were targeted when simulating age-appropriate doses for the whole pediatric age range.

**Results:**

Dexamethasone plasma concentrations upon single and multiple intravenous administration were predicted adequately across the pediatric age range. Exposure-matched predictions of dexamethasone PK indicated that doses (in mg/kg) for the 2–6 years olds can be applied in 3 month-2 year old children, whereas lower doses are needed in children of other age groups (60% lower for 0–2 weeks, 40% lower for 2–4 weeks, 20% lower for 1–3 months, 20% lower for 6–12 year olds, 40% lower for 12–18 years olds).

**Discussion:**

We show that PBPK modeling is a valuable tool that can be used to develop model-informed recommendations using dexamethasone to prevent PES in children. Based on exposure matching, the dose of dexamethasone should be reduced compared to commonly used doses, in infants <3 months and children ≥6 years, reflecting age-related variation in drug disposition. PBPK modeling is an promising tool to optimize dosing of critically ill patients.

## 1 Introduction

Pharmacotherapy is a fundamental aspect of care in the intensive care unit. Yet, due to large variation in age, size, illness severity and treatment modalities of critically ill patients, drug concentrations may vary widely (1). In children, unlicensed or off-label drug use is prevalent in pediatric ICUs (PICUs) and neonatal ICUs (NICUs), due to the lack of studies supporting the dose, effectiveness and safety (2). This leads to poorly substantiated dosing information in children, which is a significant challenge that can have serious consequences, such as suboptimal treatment or increased risk of adverse drug reactions (3). Hence, it is crucial to have evidence-based dosing guidelines tailored to the pediatric and neonatal population.

Age-related physiological changes, such as drug metabolism and renal function maturation, can significantly impact drug pharmacokinetics (PK) and thereby drug concentrations (4). These alterations necessitate age-appropriate doses to ensure optimal drug efficacy and safety. By incorporating this knowledge on age-related variation in the processes that govern disposition of drugs in pharmacokinetic models, age-appropriate dosing recommendations can be established (5). Pediatric physiologically-based pharmacokinetic (PBPK) models include age-related physiological changes and improve continuously with increasing knowledge (6). PBPK modeling is widely accepted as a promising tool to guide dosing in pediatric clinical care, as well as by regulatory agencies (US Food and Drug Administration and European Medicines Agency) for its use during pediatric drug development (7–9).

A proof of concept drug to show the value of the PBPK modeling approach to obtain age-appropriate model-informed doses is dexamethasone. Dexamethasone is standard of care in most PICUs to prevent post-extubation stridor (PES). Intubation potentially results in laryngeal injury either through the act of intubation itself or the pressure exerted by the endotracheal tube (10). Laryngeal injury can result as subglottic scaring which would require surgical intervention or as subglottic obstruction due to edema. Although laryngeal edema will often heal spontaneously after extubation, some children may develop a serious laryngeal stenosis due to edema with clinical signs of severe upper airway obstruction (11). This consequently can manifest as PES which is associated with increased morbidity due to prolonged hospital stay, risk of failed extubation, and reintubation airway trauma (12). Reported incidence of PES in children is variable, yet a recent study showed an incidence of 18.7% (13).

Dexamethasone has been shown effective to prevent PES (14, 15) by decreasing edema through its anti-inflammatory action (16). Dosing recommendations vary significantly between (hospital) guidelines, demonstrating the lack of consensus with respect to optimal dosing (17). The most commonly recommended IV dose is 0.5 mg/kg/dose for children of all ages from 1 month to 18 years old (18–21) and 0.25 mg/kg/dose for neonates (20–22). In children, guidelines recommend initiating treatment 6 to 12 h prior to extubation and then every 6 h for up to 6 doses (17, 19, 20); whereas in neonates, the first dose is often given 4 h prior to extubation, followed by a dose every 8 h for 3 doses (20). In comparison, this dose is considerably higher than the dose to prevent adverse neurological outcomes in children with meningitis (i.e., 0.15 mg/kg/dose 4 times daily) (23). Furthermore, for the treatment of acute subglottic laryngitis, characterized by a comparable disease mechanism (i.e., subglottic laryngeal edema), a dose of 0.15 mg/kg once is as effective as 0.6 mg/kg (24, 25). Hence, we consider that a more optimal (reduced) dose might be appropriate to prevent PES and current recommendations [i.e., 0.5 mg/kg for children >1 month of age (19)] may arguably exceed the necessary dosage for effective prevention of PES. Additionally, high dexamethasone doses have been associated with adverse effects such as hypertension (26) and corticosteroid exposure in preterm infants has been associated with adverse neurological outcomes (27).

Dexamethasone is primarily metabolized by the drug metabolizing enzyme cytochrome P450 (CYP)3A4 of which the activity increases rapidly in neonates to reach a maximum in infants and young children, to decrease to adult levels thereafter (28). Hence, CYP3A4 activity will substantially affect dexamethasone clearance and thus its plasma concentration across the pediatric age span. This is currently not reflected in dosing recommendations, risking over- or underdosing with corresponding toxicity or therapy failure, respectively. Therefore, dexamethasone dosing recommendations could be optimized taking into account CYP3A4 ontogeny. Establishing dosing recommendations based on exposure matching is an acceptable approach assuming that exposure-response relationships are similar between populations (7–9). We have previously demonstrated that a pragmatic PBPK modeling approach is feasible and described the workflow in detail (29, 30). The objective of this study is to develop age-appropriate dosing recommendations for children of dexamethasone for PES, as proof of concept for PBPK modeling to individualize dosing for critically ill patients.

## 2 Material and methods

### 2.1 Pediatric dexamethasone PBPK model verification

To conduct PBPK simulations, we used Simcyp™ v21 (Certara UK Limited, Simcyp Division, Sheffield, UK), a population-based PBPK modeling platform. The software already contains a well-validated pediatric population model with age-related varying physiology, including CYP3A4 ontogeny (31). This population model was linked to a dexamethasone model containing all drug-specific properties to predict dexamethasone PK in children (Supplementary Table S2). To verify that this model adequately predicts dexamethasone concentrations in children across the pediatric age span, published pediatric PK data were searched first to compare predicted dexamethasone concentrations with these observed data (Supplementary Table S1). Accuracy of model predictions (i.e., predictive performance) was assessed quantitatively by calculating predicted-to-observed PK parameter ratios (within 2-fold was considered acceptable) and qualitatively by a visual predictive check of the comparison between the predicted and the observed plasma concentration-time curves. A more detailed description of the model verification process can be found in the Supplementary Materials.

### 2.2 Dose simulations

Next, to apply the model and simulate optimized dexamethasone doses, we first had to select an exposure margin, i.e., target concentrations, that ensures an effective and safe dose. Explicit effective and safe concentrations (i.e., a therapeutic window or PK target) for this indication are unknown. To overcome this information gap, we first searched the literature for doses that have been shown effective in children. Several dosing schedules were included: a “low early” regimen with 6 IV administrations of 0.25 mg/kg every 6 h (q6h) (initiated >24 h prior to extubation), and a “high late” regimen with 3 IV administrations of 0.5 mg/kg IV doses q6h (initiated 6–12 h prior to extubation) (32). As subglottic laryngitis has a comparable disease mechanism, we considered that the dose recommended for this condition (0.15 mg/kg) could also be effective to prevent PES. All dosing strategies apply a maximum of 40 mg/day. Moreover, in one of the involved PICUs (i.e., Erasmus MC), a similar dose is already applied to prevent PES. Therefore, also a doubled recommended dose for acute subglottic laryngitis is included in the analysis, meaning administrating twice 0.15 mg/kg IV 6 h apart (33).

As evidence for efficacy was strongest in the 2–6 year old age group (i.e., “the best-evidence age group”), we used the simulated total exposure for this age group as the effective target exposure to aim for when simulating age-appropriate doses for the whole pediatric age range. In other words, the total exposures over 48 h (area under the curve; AUC_0–48_) of the different pediatric age ranges (i.e., 0–2, 2–4 weeks, 1–3, 3–6, 6–12 months, 1–2, 6–12, and 12–18 years) were matched to the effective target exposure of “the best-evidence age group”. Establishing dosing recommendations based on exposure matching is an acceptable approach assuming exposure-response relationships are similar across the pediatric age range (7–9). Additionally, the current dosing regimens from the Dutch Pediatric Formulary for prophylaxis of PES were simulated for comparison (Supplementary Table S3) (21).

## 3 Results

**Figure 1:**
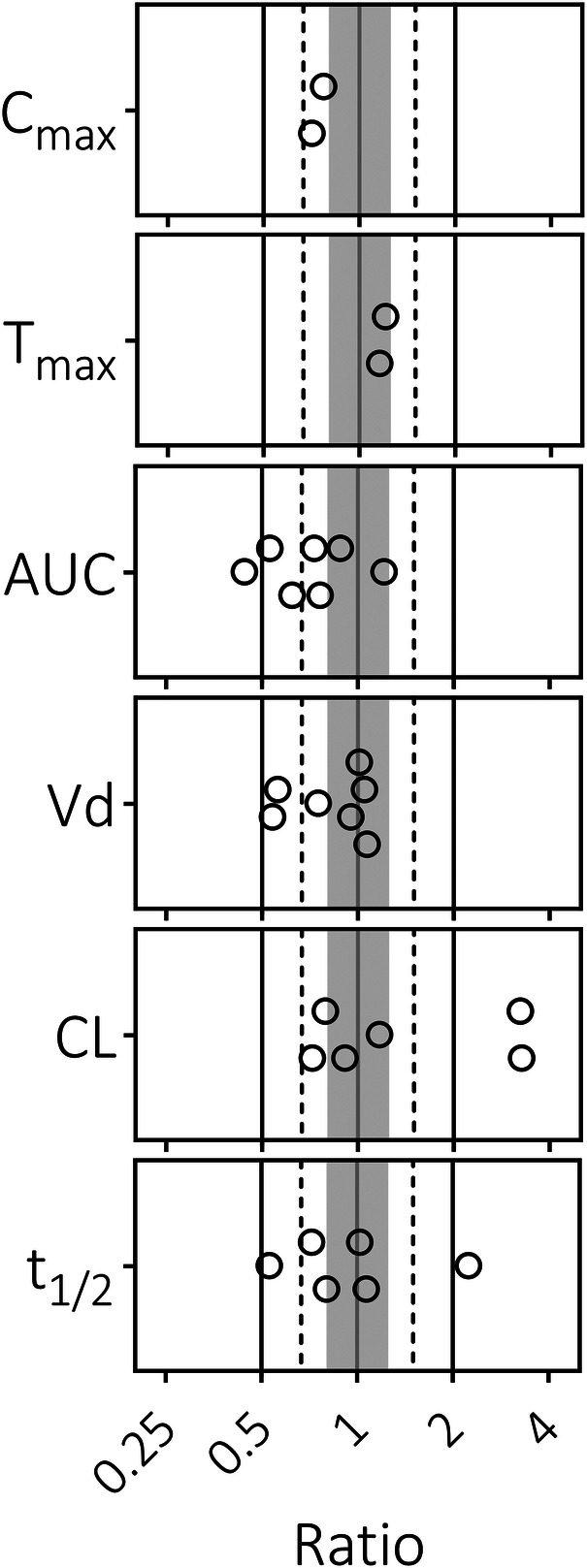
Predicted-to-observed ratios of dexamethasone pharmacokinetic parameters in pediatrics. Maximum concentration (C_max_), time to peak concentration (T_max_), area under the curve (AUC), volume of distribution (Vd), clearance (CL), and half-life (t_1/2_). Single symbols represent a predicted-to-observed ratio of a single pharmacokinetic study. Included pediatric age range is 0.33–18.8 years. The black lines represent the 2-fold range, the dashed lines the 1.5-fold range, the gray shaded area represents the 1.25-fold range and the gray line represents the unity line.

**Figure 2:**
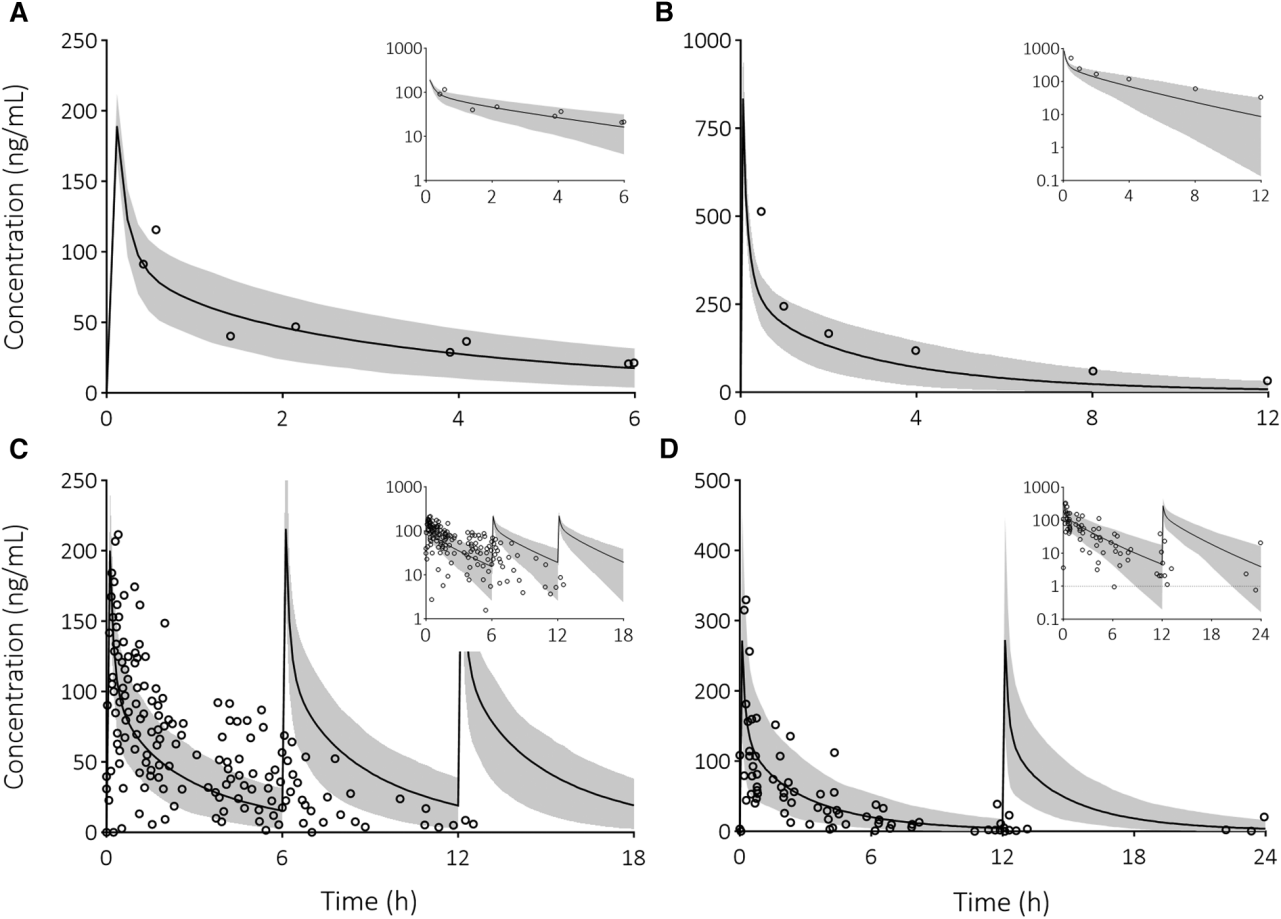
Visual predictive checks of predicted dexamethasone plasma concentration-time profiles compared to observed clinical data in pediatric patients. Solid line is the predicted mean and the shaded area represents the 5th–95th percentile of the predicted plasma concentration in the virtual population. Open circles are the observed data after the following IV doses: (**A**) 3 mg/m^2^ dose (34), (**B**) 0.3 mg/kg dose (35), (**C**) 3 mg/m^2^ every 6 h (36), and (**D**) 4 mg every 12 h (36). Insets provide the results on a semi-logarithmic scale. The dashed line indicates the lower limit of quantification.

To determine whether the pediatric PBPK model can accurately predict dexamethasone concentrations, we compared dexamethasone PK predictions with observed data. The majority of predicted-to-observed PK parameter ratios fall within the 2-fold range (Figure 1). Furthermore, the predicted dexamethasone plasma concentrations over time upon single (Figures 2A,B) and multiple (Figures 2C,D) IV administrations are in good agreement with corresponding observed concentrations. Both assessments indicate, quantitively as well as qualitatively, that the model predicts dexamethasone concentrations adequately across the pediatric age range.

**Figure 3:**
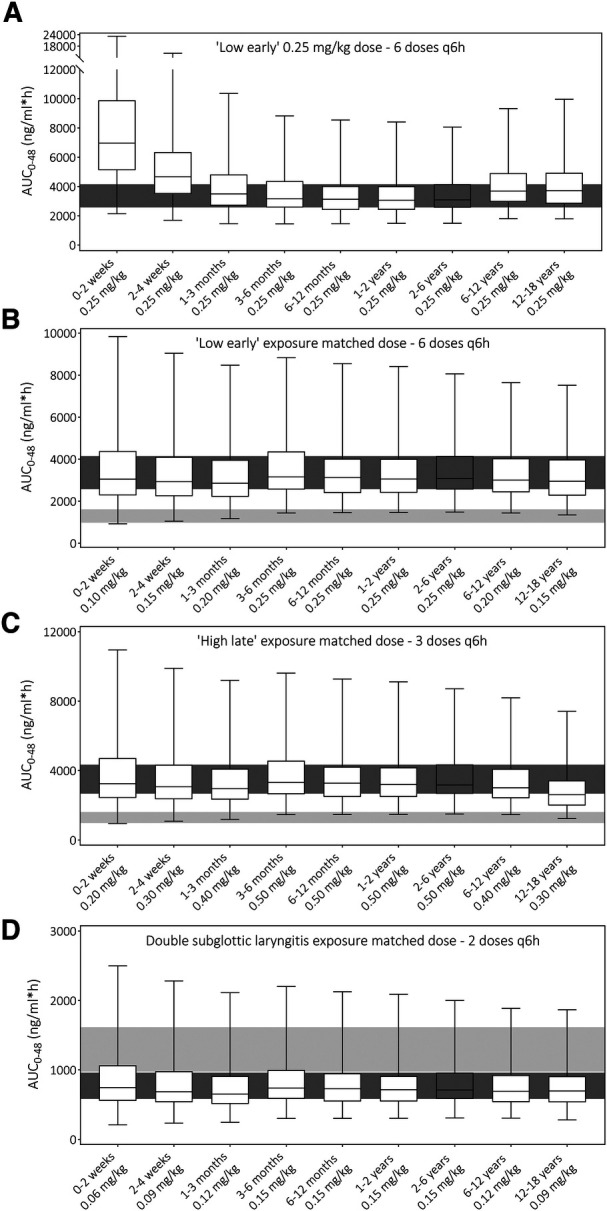
Prediction of dexamethasone total exposure over 48 h (AUC_0−48_) following different dosing schedules (**A**) unmatched exposure upon “Low early” dosing (32), (**B**) exposure matched “Low early” dosing (32), (**C**) exposure matched “High late” dosing (32), and (**D**) exposure matched double subglottic laryngitis dose (33). Boxes indicate the median prediction with the 25th and 75th percentile ranges, while the whiskers indicate the predicted min and max values of the simulated populations. The dark gray shaded area indicates the 25th–75th percentile range of the 2–6 years age group prediction to which other exposures are matched. The light gray shaded area is the predicted 25th–75th percentile range of total exposure with the current Dutch Paediatric Formulary recommendation in the 2–6 years age group (21).

After model verification, varying dosing scenarios were simulated in pediatric age groups to determine exposure with the currently used as well as optimized dosing schedules. Figure 3A illustrates this, showing predicted total drug exposures when using the same dose for all age groups from the dosing scenarios “Low early” (i.e., unmatched to exposures). It indicates a considerably higher exposure in neonates <1 month of age. Next, Figures 3B–D show exposures for the 2–6 years age group as well as the final model-informed dosing recommendations for all age groups to match the exposure from the 2–6 years of age following the dosing scenarios “Low early”, “High late”, and “double subglottic laryngitis”. To match these exposures in the other age groups, body-weight normalized dose adjustments were needed as follows: no dosing adjustments for 3 months-2 years of age (i.e., 100%), 80% of the original dose for the age groups 1–3 months (e.g., 0.4 vs. 0.5 mg/kg), 60% for the age groups 2–4 weeks and 12–18 years (e.g., 0.3 vs. 0.5 mg/kg), and 40% for the 0–2 weeks age group (e.g., 0.2 vs. 0.5 mg/kg). Current dosing recommendations from varying guidelines and all model-informed doses per age group, per dosing scenario are provided in Table 1.

**Table 1:** Current and proposed model-informed dosing regimens for prophylaxis of post-extubation stridor.

	Age group	AAP (19)	Dutch Pediatric formulary (21)	Harriet Lane Handbook (37)	Leicester Children's Hospital (38)	Lexicomp (20)	Pediatric Ventilator Liberation Guideline (17)
Current doses	Neonates	–	0.25 mg/kg/dose for 3 doses start 4 h prior, 8 and 16 h post	–	–	0.25 mg/kg/dose for 3 doses start 4 h prior, 8 and 16 h post (max 1.5 mg/kg/day)	–
	1 month–18 years	0.5 mg/kg/dose q6h for 6 doses start 6–12 h prior (max 10 mg/dose)	0.5 mg/kg/dose once, repeat if necessary (max 40 mg/day)	0.5–2 mg/kg/day q6h for 4–6 doses start 24 h prior	0.2 mg/kg q6h for 6 doses start 6–12 h prior (max 10 mg/dose)	0.5 mg/kg/dose q6h for 6 doses start 6–12 h prior (max 10 mg/dose)	0.15–0.5 mg/kg/dose, start >6 h prior (ideally 12–24 h). If within 6 h, 0.5 mg/kg/dose should be considered (max 10 mg)
	Age group		“Low early” Start 12–24 h prior 6x q6h*		“High late” Start 6–12 h prior 3x q6h*		“Subglottic laryngitis” Start 1–6 h prior 2x q6h*
Proposed model-informed doses	Term neonates (0–2 weeks)		0.10 mg/kg		0.20 mg/kg		0.06 mg/kg
	Term neonates (2–4 weeks)		0.15 mg/kg		0.30 mg/kg		0.09 mg/kg
	1–3 months		0.20 mg/kg		0.40 mg/kg		0.12 mg/kg
	3 months–6 years		0.25 mg/kg		0.50 mg/kg		0.15 mg/kg
	6–12 years		0.20 mg/kg		0.40 mg/kg		0.12 mg/kg
	12–18 years		0.15 mg/kg		0.30 mg/kg		0.09 mg/kg

* >1 month all max 40 mg/day.

## 4 Discussion

In this study, we applied “pragmatic” PBPK modeling and simulation to establish age-appropriate dosing recommendations for IV dexamethasone to prevent PES in children. By simulating dosing regimens in pediatric populations with small age ranges, we established tailor-made, model-informed doses, especially taking into account the CYP3A4 maturation as well as other age-related physiological changes. Dosing simulations indicate that a significant reduced dose is required in neonates compared to children aged 3 months to 6 years, i.e., 40%–60% of the original dose. To note, the proposed dosing recommendations are off-label as dexamethasone is not registered for prevention of PES as an indication. We hereby show the successful use of “pragmatic” PBPK modeling to optimize dosing in the ICU setting.

The model-informed dosing approach has been employed successfully in pediatric drug development, to establish first-in-child doses for clinical trials, and is approved by regulatory agencies (i.e., EMA and FDA). Lately, PBPK dose simulations have also been used to establish pediatric doses for direct use in clinical care (39, 40). During the early stage of the COVID-19 pandemic, this approach was taken to quickly provide healthcare practitioners with pediatric doses for chloroquine and hydroxychloroquine (although now obsolete for COVID-19) (41, 42). Applying existing PBPK models in a pediatric setting minimizes the need for PK studies as PBPK simulations can quickly inform dosing for clinical practice (5, 43). We have recently described the opportunities and challenges to apply “pragmatic” PBPK modeling to establish pediatric drug doses (29, 30). Here, we successfully employed this approach to accurately predict dexamethasone exposures in children. We established moderate, yet important, impact model-informed doses in which the maturation and ontogeny of involved distribution, metabolism, and elimination processes are well-characterized and the relative contribution of elimination pathways is incorporated accurately (i.e., CYP3A4 metabolism) across the whole pediatric age range (30). In addition to PBPK modeling, population PK (popPK) models can also be considered for deriving model-informed dosing recommendations, such as with ceftriaxone and cefotaxime for severe infections (44, 45). How to develop popPK-based model-informed dosing guidelines for clinical implementation has been addressed in an earlier published framework (46). PopPk models and consequent dosing simulations are based on available pharmacokinetic data from the target population. In contrast, PBPK models incorporate human physiology and drug data, and can describe pharmacokinetics and can be used for dose simulations with sparse or even no pharmacokinetic data at all. In this paper we used the PBPK approach to simulate optimal dexamethasone dosing.

Knowledge of an effective and safe exposure target is required to apply the established model to determine which dose results in the desired target exposure. Although evidence to support efficacy for dexamethasone to prevent PES in children is relatively limited and uniform dosing guidance is missing, its use is standard of care in most PICUs (21, 37, 38). Still, well-known dosing guidelines advice a similar body weight-adjusted dose across the pediatric age range from 1 month up until the age of 18 years. From a pharmacological perspective, this is suboptimal because the developmental changes in the physiological processes that govern drug disposition may lead to under- or overdosing in children of different ages, with an increased risk of unfavorable clinical effects. As a therapeutic window or therapeutic target is not established for dexamethasone to prevent PES, we could not use this information to simulate age-appropriate doses. As the best alternative, a best-evidence target range was established by taking the best studied age group (i.e., 2–6 years) and its effective dose as a starting point. As not one single dosing regimen has been unequivocally shown to be most effective, we simulated several “best-evidence” dosing schedules for which relative strong evidence for effectiveness was available (32), in addition to a doubled recommended dose for subglottic laryngitis (33) to provide insights with multiple therapeutic targets. The use of dexamethasone to treat subglottic laryngitis is well established and a single low dose (i.e., 0.15 mg/kg) is shown to be effective (25). The disease mechanisms of PES and subglottic laryngitis show similarities, as both result in laryngeal edema. Though, relatively weak evidence shows that comparable low dexamethasone doses to prevent PES seem ineffective (26, 47). It has been argued that although PES and subglottic laryngitis manifest in a similar manner, the difference in etiology and mechanism of cellular damage may explain failure of PES treatment with low dexamethasone doses (47). Still, it is included in our study as a similar dose is already applied in one of the involved PICUs (i.e., Erasmus MC) to prevent PES. Model-informed doses may be implemented into clinical guidelines, despite the lack of prospective validation. The current doses, particularly for less studied age groups, have not been proven effective or safe and are likely too high, posing a risk of toxicity. We believe it is more ethical to adjust current doses based on robust pharmacokinetic and developmental physiological knowledge, as well as evidence from well-studied age groups. While, at the same time we call for efficacy studies to finally support our model-informed doses. In case physicians are hesitant to implement our proposed doses, an effectiveness-implementation hybrid study is suggested to confirm the effectiveness where parents can make an informed decision choosing between the standard of care or the reduced proposed doses (48).

This study has its limitations as well. Firstly, the ideal timing of treatment initiation prior to extubation is not taken into account by the model. Iyer and colleagues demonstrated in a network meta-analysis that early initiation (>12 h) of low dose dexamethasone intervention, i.e., < 0.5 mg/kg/dose, is as effective as early initiation of high doses (≥0.5 mg/kg/dose) to prevent PES (14). Furthermore, a subgroup analysis in adults revealed that PES could be reduced when corticosteroids were administered as multiple doses and initiated 12–24 h prior to extubation compared to single doses closer to extubation (18). No such comparison has been made with pediatric patients. Regardless of the evidence suggesting that early initiation of dexamethasone treatment is beneficial, it is difficult to anticipate if a patient is ready for extubation 12 or more hours prior to the procedure. The patient may deteriorate in the meantime, resulting in postponing extubation and hence unnecessary dexamethasone treatment (i.e., cumulative overdosing), which is undesirable as well. Thus, as apparent evidence is lacking on the most optimal dosing schedule (timing of first dose and number of repeated doses), we do not provide absolute dosing guidelines, instead we propose guidance to proportionally adjust doses based on age.

Secondly, published dexamethasone PK data are only available in pediatric populations with a wide age range (e.g., 1 month–18 years) making it challenging to assess model performance with high confidence in age groups that are small with respect to age range. Yet, for midazolam, also a mainly CYP3A4 metabolized drug, model performance has been evaluated in younger and more specific age groups as such clinical PK data are available for midazolam (49). This improves the confidence in our model to accurately predict dexamethasone plasma concentrations.

Thirdly, we simulated a healthy pediatric population. Though, pediatric patients requiring mechanical ventilation in the ICU are considered critically ill, often with different underlying conditions (50), that can considerably impact PK (51). It has been identified previously that inflammation and organ failure significantly reduce the clearance of CYP3A4-metabolized drugs (i.e., midazolam a CYP3A4 probe drug) (52, 53). Strong *in vitro* evidence has shown that CYP3A4 is downregulated by the inflammatory cytokines interleukin-6 and interleukin-1β (54). Although most information regarding the impact of critical illness on drug clearance is available for other CYP3A4-metabolized drugs, such as midazolam, a similar disease-drug effect can be expected for dexamethasone. Since we compared exposure across the pediatric age range for a similar population (PICU patients ready for extubation), we do not expect that our “proportional” dose advice is strongly affected by the impact of critical illness, as the levels of critical illness are likely similar at extubation. On the contrary, our proposed dosing adjustments for PES are in theory extrapolatable to other dexamethasone indications when efficacy may be determined via matched exposures, such as the use of dexamethasone to prevent nausea and vomiting during chemotherapy. Additionally, while extensive pharmacokinetic data confirm similar age-related variation for other CYP3A4 metabolized drugs, such as midazolam, quinidine and tacrolimus, this knowledge has only minimally been implemented in age-appropriate doses for clinical use (19, 20, 55). Our proportional dosing advice may also apply to these drugs and it is therefore interesting to evaluate if more appropriate doses can be established for other CYP3A4 metabolized drugs as well.

## 5 Conclusion

In conclusion, we show that PBPK modeling is a valuable tool that can be used to develop model-informed age-appropriate dosing recommendations in the ICU setting, with dexamethasone to prevent PES in children as a proof of concept. Our data indicate that, based on exposure matching, the weight-based dose of dexamethasone should be lower in the youngest and oldest age groups compared to children between 2 and 6 years of age. The use of PBPK modeling and, here, extrapolation of efficacy through exposure matching, negates the need for extensive prospective pharmacokinetic and/or dose-finding studies, yet provides valuable comprehensive evidence to inform clinical practice and potentially pediatric drug labeling.

## Data Availability

The raw data supporting the conclusions of this article will be made available by the authors, without undue reservation.

## References

[1] Morales Castro D Dresser L Granton J Fan E. Pharmacokinetic alterations associated with critical illness. Clin Pharmacokinet. (2023) 62(2):209–20. 10.1007/s40262-023-01213-x 36732476 PMC9894673

[2] Nir-Neuman H Abu-Kishk I Toledano M Heyman E Ziv-Baran T Berkovitch M. Unlicensed and off-label medication use in pediatric and neonatal intensive care units: no change over a decade. Adv Ther. (2018) 35(7):1122–32. 10.1007/s12325-018-0732-y 29949042

[3] Bellis JR Kirkham JJ Thiesen S Conroy EJ Bracken LE Mannix HL Adverse drug reactions and off-label and unlicensed medicines in children: a nested case-control study of inpatients in a pediatric hospital. BMC Med. (2013) 11:238. 10.1186/1741-7015-11-238 24229060 PMC4231613

[4] Kearns GL Abdel-Rahman SM Alander SW Blowey DL Leeder JS Kauffman RE. Developmental pharmacology–drug disposition, action, and therapy in infants and children. N Engl J Med. (2003) 349(12):1157–67. 10.1056/NEJMra035092 13679531

[5] Freriksen JJM van der Heijden JEM de Hoop-Sommen MA Greupink R de Wildt SN. Physiologically based pharmacokinetic (PBPK) model-informed dosing guidelines for pediatric clinical care: a pragmatic approach for a special population. Paediatr Drugs. (2023) 25(1):5–11. 10.1007/s40272-022-00535-w 36201128 PMC9534738

[6] Verscheijden LFM Koenderink JB Johnson TN de Wildt SN Russel FGM. Physiologically-based pharmacokinetic models for children: starting to reach maturation? Pharmacol Ther. (2020) 211:107541. 10.1016/j.pharmthera.2020.107541 32246949

[7] Mulugeta Y Barrett JS Nelson R Eshete AT Mushtaq A Yao L Exposure matching for extrapolation of efficacy in pediatric drug development. J Clin Pharmacol. (2016) 56(11):1326–34. 10.1002/jcph.744 27040726 PMC5482171

[8] Faraj A van Wijk RC Neuman L Desai S Blouse GE Knudsen T Model-informed pediatric dose selection of marzeptacog alfa (activated): an exposure matching strategy. CPT Pharmacometrics Syst Pharmacol. (2023) 12(7):977–87. 10.1002/psp4.12967 37042339 PMC10349190

[9] Jo H Pilla Reddy V Parkinson J Boulton DW Tang W. Model-Informed pediatric dose selection for dapagliflozin by incorporating developmental changes. CPT Pharmacometrics Syst Pharmacol. (2021) 10(2):108–18. 10.1002/psp4.12577 33439535 PMC7894404

[10] Duynstee ML de Krijger RR Monnier P Verwoerd CD Verwoerd-Verhoef HL. Subglottic stenosis after endolaryngeal intubation in infants and children: result of wound healing processes. Int J Pediatr Otorhinolaryngol. (2002) 62(1):1–9. 10.1016/S0165-5876(01)00545-6 11738687

[11] Weiss M Dave M Bailey M Gysin C Hoeve H Hammer J Endoscopic airway findings in children with or without prior endotracheal intubation. Paediatr Anaesth. (2013) 23(2):103–10. 10.1111/pan.12102 23289772

[12] Shinohara M Iwashita M Abe T Takeuchi I. Risk factors associated with symptoms of post-extubation upper airway obstruction in the emergency setting. J Int Med Res. (2020) 48(5):300060520926367. 10.1177/0300060520926367 32468931 PMC7263151

[13] Veder LL Joosten KFM Schlink K Timmerman MK Hoeve LJ van der Schroeff MP Post-extubation stridor after prolonged intubation in the pediatric intensive care unit (PICU): a prospective observational cohort study. Eur Arch Otorhinolaryngol. (2020) 277(6):1725–31. 10.1007/s00405-020-05877-0 32130509 PMC7198633

[14] Iyer NP López-Fernández YM González-Dambrauskas S Baranwal AK Hotz JC Zhu M A network meta-analysis of dexamethasone for preventing postextubation upper airway obstruction in children. Ann Am Thorac Soc. (2023) 20(1):118–30. 10.1513/AnnalsATS.202203-212OC 35976878 PMC9819263

[15] Butragueño-Laiseca L Manrique Martín G González Cortés R Rey Galán C Martínez de Compañón Martínez de Marigorta Z Antón JG Multicenter randomized clinical trial comparing dexamethasone versus placebo in preventing upper airway obstruction after extubation in critically ill children. Sci Rep. (2022) 12(1):4336. 10.1038/s41598-022-08178-0 35288599 PMC8921236

[16] Rhen T Cidlowski JA. Antiinflammatory action of glucocorticoids–new mechanisms for old drugs. N Engl J Med. (2005) 353(16):1711–23. 10.1056/NEJMra050541 16236742

[17] Abu-Sultaneh S Iyer NP Fernández A Gaies M González-Dambrauskas S Hotz JC Executive summary: international clinical practice guidelines for pediatric ventilator liberation, A pediatric acute lung injury and sepsis investigators (PALISI) network document. Am J Respir Crit Care Med. (2023) 207(1):17–28. 10.1164/rccm.202204-0795SO 36583619 PMC9952867

[18] Khemani RG Randolph A Markovitz B. Corticosteroids for the prevention and treatment of post-extubation stridor in neonates, children and adults. Cochrane Database Syst Rev. (2009) 2009(3):Cd001000. 10.1002/14651858.CD001000.pub3 19588321 PMC7096779

[19] Shenoi RP Timm N, DRUGS CO, MEDICINE COPE, Jones B Neville K Drugs used to treat pediatric emergencies. Pediatrics. (2020) 145(1):e20193450. 10.1542/peds.2019-3450 31871244

[20] Lexicomp Online, Pediatric and Neonatal Lexi-Drugs Online. Available online at: https://online.lexi.com (updated July 25–26, 2023).

[21] Kinderformularium. Kinderformularium – Dexamethason. Available online at: https://www.kinderformularium.nl/geneesmiddel/182/dexamethason (updated July 12, 20190) (cited May 10, 2023).

[22] Davis PG Henderson-Smart DJ. Intravenous dexamethasone for extubation of newborn infants. Cochrane Database Syst Rev. (2001) 4:Cd000308. 10.1002/14651858.CD000308 11687075

[23] Brouwer MC McIntyre P Prasad K van de Beek D. Corticosteroids for acute bacterial meningitis. Cochrane Database Syst Rev. (2015) 2015(9):Cd004405. 10.1002/14651858.CD004405.pub5 26362566 PMC6491272

[24] Geelhoed GC Macdonald WB. Oral dexamethasone in the treatment of croup: 0.15 mg/kg versus 0.3 mg/kg versus 0.6 mg/kg. Pediatr Pulmonol. (1995) 20(6):362–8. 10.1002/ppul.1950200605 8649915

[25] Chub-Uppakarn S Sangsupawanich P. A randomized comparison of dexamethasone 0.15 mg/kg versus 0.6 mg/kg for the treatment of moderate to severe croup. Int J Pediatr Otorhinolaryngol. (2007) 71(3):473–7. 10.1016/j.ijporl.2006.11.016 17208307

[26] Ritu, Jhamb U. Dexamethasone in prevention of postextubation stridor in ventilated children: a randomized, double-blinded, placebo-controlled trial. Indian J Crit Care Med. (2020) 24(12):1230–5. 10.5005/jp-journals-10071-23679 33446978 PMC7775922

[27] Räikkönen K Gissler M Kajantie E. Associations between maternal antenatal corticosteroid treatment and mental and behavioral disorders in children. Jama. (2020) 323(19):1924–33. 10.1001/jama.2020.3937 32427304 PMC7237984

[28] Upreti VV Wahlstrom JL. Meta-analysis of hepatic cytochrome P450 ontogeny to underwrite the prediction of pediatric pharmacokinetics using physiologically based pharmacokinetic modeling. J Clin Pharmacol. (2016) 56(3):266–83. 10.1002/jcph.585 26139104

[29] van der Heijden JEM Freriksen JJM de Hoop-Sommen MA van Bussel LPM Driessen SHP Orlebeke AEM Feasibility of a pragmatic PBPK modeling approach: towards model-informed dosing in pediatric clinical care. Clin Pharmacokinet. (2022) 61(12):1705–17. 10.1007/s40262-022-01181-8 36369327 PMC9651907

[30] van der Heijden JEM Freriksen JJM de Hoop-Sommen MA Greupink R de Wildt SN. PBPK modeling for drug dosing in pediatric patients: a tutorial for a pragmatic approach in clinical care. Clin Pharmacol Ther. (2023) 114(5):960–71. 10.1002/cpt.3023 37553784

[31] Johnson TN Rostami-Hodjegan A Tucker GT. Prediction of the clearance of eleven drugs and associated variability in neonates, infants and children. Clin Pharmacokinet. (2006) 45(9):931–56. 10.2165/00003088-200645090-00005 16928154

[32] Parajuli B Baranwal AK Kumar MP Jayashree M Takia L. Twenty-four-hour pretreatment with low dose (0.25 mg/kg/dose) versus high dose (0.5 mg/kg/dose) dexamethasone in reducing the risk of postextubation airway obstruction in children: a randomized open-label noninferiority trial. Pediatr Pulmonol. (2021) 56(7):2292–301. 10.1002/ppul.25388 33764654

[33] Pediatric Association of the Netherlands (Nederlandse vereniging voor Kindergeneeskunde). Dexamethason bij Laringitis Subglottica. Available online at: http://www.nvk.nl/ (updated July 28, 2014) (cited August 1, 2023).

[34] Nijstad AL Tibben MM Gebretensae A Rosing H de Vos-Kerkhof E Zwaan CM Development and validation of a combined liquid chromatography tandem-mass spectrometry assay for the quantification of aprepitant and dexamethasone in human plasma to support pharmacokinetic studies in pediatric patients. J Chromatogr B Analyt Technol Biomed Life Sci. (2021) 1171:122639. 10.1016/j.jchromb.2021.122639 33756449

[35] Richter O Ern B Reinhardt D Becker B. Pharmacokinetics of dexamethasone in children. Pediatr Pharmacol (New York). (1983) 3(3–4):329–37. 6677878

[36] Nijstad AL de Vos-Kerkhof E Enters-Weijnen CF van de Wetering MD Tissing WJE Tibben MM Overestimation of the effect of (fos)aprepitant on intravenous dexamethasone pharmacokinetics requires adaptation of the guidelines for children with chemotherapy-induced nausea and vomiting. Support Care Cancer. (2022) 30(12):9991–9. 10.1007/s00520-022-07423-6 36287279 PMC9607815

[37] Service HL Hughes HK Kahl L. The Harriet Lane Handbook: A Manual for Pediatric House Officers. Philadelphia: Elsevier (2018).

[38] Westrope C. Leicester Children’s Hospital PICU guideline: Pevention and management of Post Extubation Stridor (2021). Available online at: https://secure.library.leicestershospitals.nhs.uk/PAGL/Shared%20Documents/Post%20Extubation%20Stridor%20UHL%20Paediatric%20Intensive%20Care%20Guideline.pdf (Accessed August 1, 2023).

[39] de Hoop-Sommen MA van der Heijden JEM Freriksen JJM Greupink R de Wildt SN. Pragmatic physiologically-based pharmacokinetic modeling to support clinical implementation of optimized gentamicin dosing in term neonates and infants: proof-of-concept. Front Pediatr. (2023) 11:1288376. 10.3389/fped.2023.1288376 38078320 PMC10702772

[40] Jacobs TG de Hoop-Sommen MA Nieuwenstein T van der Heijden JEM de Wildt SN Burger DM Lamivudine and emtricitabine dosing proposal for children with HIV and chronic kidney disease, supported by physiologically based pharmacokinetic modelling. Pharmaceutics. (2023) 15(5):1424. 10.3390/pharmaceutics15051424 37242665 PMC10221211

[41] Verscheijden LFM van der Zanden TM van Bussel LPM de Hoop-Sommen M Russel FGM Johnson TN Chloroquine dosing recommendations for pediatric COVID-19 supported by modeling and simulation. Clin Pharmacol Ther. (2020) 108(2):248–52. 10.1002/cpt.1864 32320477 PMC7264731

[42] Maharaj AR Wu H Hornik CP Balevic SJ Hornik CD Smith PB Simulated assessment of pharmacokinetically guided dosing for investigational treatments of pediatric patients with coronavirus disease 2019. JAMA Pediatr. (2020) 174(10):e202422. 10.1001/jamapediatrics.2020.2422 32501511 PMC7275264

[43] Dunne J Rodriguez WJ Murphy MD Beasley BN Burckart GJ Filie JD Extrapolation of adult data and other data in pediatric drug-development programs. Pediatrics. (2011) 128(5):e1242–9. 10.1542/peds.2010-3487 22025597

[44] Hartman SJF Upadhyay PJ Hagedoorn NN Mathôt RAA Moll HA van der Flier M Current ceftriaxone dose recommendations are adequate for most critically ill children: results of a population pharmacokinetic modeling and simulation study. Clin Pharmacokinet. (2021) 60(10):1361–72. 10.1007/s40262-021-01035-9 34036552 PMC8505376

[45] Hartman SJF Upadhyay PJ Mathôt RAA van der Flier M Schreuder MF Brüggemann RJ Population pharmacokinetics of intravenous cefotaxime indicates that higher doses are required for critically ill children. J Antimicrob Chemother. (2022) 77(6):1725–32. 10.1093/jac/dkac095 35383374 PMC9155601

[46] Hartman SJF Swaving JGE van Beek SW van Groen BD de Hoop M van der Zanden TM A new framework to implement model-informed dosing in clinical guidelines: piperacillin and amikacin as proof of concept. Front Pharmacol. (2020) 11:592204. 10.3389/fphar.2020.592204 33390970 PMC7772249

[47] Cesar RG de Carvalho WB. L-epinephrine and dexamethasone in postextubation airway obstruction: a prospective, randomized, double-blind placebo-controlled study. Int J Pediatr Otorhinolaryngol. (2009) 73(12):1639–43. 10.1016/j.ijporl.2009.08.004 19762088

[48] Curran GM Bauer M Mittman B Pyne JM Stetler C. Effectiveness-implementation hybrid designs: combining elements of clinical effectiveness and implementation research to enhance public health impact. Med Care. (2012) 50(3):217–26. 10.1097/MLR.0b013e3182408812 22310560 PMC3731143

[49] Johnson TN Howgate EM de Wildt SN Turner MA Rowland Yeo K. Use of developmental midazolam and 1-hydroxymidazolam data with pediatric physiologically based modeling to assess cytochrome P450 3A4 and uridine diphosphate glucuronosyl transferase 2B4 ontogeny *in vivo*. Drug Metab Dispos. (2023) 51(8):1035–45. 10.1124/dmd.123.001270 37169511

[50] Fraser J Henrichsen T Mok Q Tasker RC. Prolonged mechanical ventilation as a consequence of acute illness. Arch Dis Child. (1998) 78(3):253. 10.1136/adc.78.3.253 9613357 PMC1717488

[51] Zuppa AF Barrett JS. Pharmacokinetics and pharmacodynamics in the critically ill child. Pediatr Clin North Am. (2008) 55(3):735–55. xii. 10.1016/j.pcl.2008.02.017 18501763

[52] Vet NJ Brussee JM de Hoog M Mooij MG Verlaat CW Jerchel IS Inflammation and organ failure severely affect midazolam clearance in critically ill children. Am J Respir Crit Care Med. (2016) 194(1):58–66. 10.1164/rccm.201510-2114OC 26796541

[53] Brussee JM Vet NJ Krekels EHJ Valkenburg AJ Jacqz-Aigrain E van Gerven JMA Predicting CYP3A-mediated midazolam metabolism in critically ill neonates, infants, children and adults with inflammation and organ failure. Br J Clin Pharmacol. (2018) 84(2):358–68. 10.1111/bcp.13459 29072785 PMC5777436

[54] Dunvald AD Järvinen E Mortensen C Stage TB. Clinical and molecular perspectives on inflammation-mediated regulation of drug metabolism and transport. Clin Pharmacol Ther. (2022) 112(2):277–90. 10.1002/cpt.2432 34605009

[55] Völler S Flint RB Beggah F Reiss I Andriessen P Zimmermann LJI Recently registered midazolam doses for preterm neonates do not lead to equal exposure: a population pharmacokinetic model. J Clin Pharmacol. (2019) 59(10):1300–8. 10.1002/jcph.1429 31093992 PMC6767398

