# Supplementary material for: Getting the dose right using physiologically-based pharmacokinetic modeling: dexamethasone to prevent post-extubation stridor in children as proof of concept
Source: Front Pediatr. 2024 Jul 5;12:1416440. doi: 10.3389/fped.2024.1416440 (PMC11257885; doi:10.3389/fped.2024.1416440)
Supplement: Supplementary file 1 [file Datasheet1.pdf]

Supplementary Material

Getting the dose right using physiologically-based pharmacokinetic modeling: dexamethasone to prevent post-extubation stridor in children as proof of concept.

Joyce E.M. van der Heijden[1], Marika de Hoop-Sommen[1], Noa Hoevenaars[1], Jolien J.M. Freriksen[1], Koen Joosten[2], Rick Greupink[1], Saskia N. de Wildt[1,2]

[1]Division of Pharmacology and Toxicology, Department of Pharmacy, Radboud university medical center, Nijmegen, The Netherlands; [2]Department of Neonatal and Pediatric Intensive Care, Division of Pediatric Intensive care, Erasmus MC-Sophia Children's Hospital, Rotterdam, The Netherlands

Corresponding author – Joyce E.M. van der Heijden, Joyce.vanderHeijden@radboudumc.nl.

Content

1. Search strategy 2
2. Pharmacokinetic studies 3
3. Model input parameters 4
4. Model verification 6
5. Simulating alternative dosing regimens 15
6. References 21

1. Search strategy

The PubMed database was searched for dexamethasone pediatric pharmacokinetic data. Both the drugname and brandname were included in the search query. Titles and abstracts of all search results were screened to check if actual PK data were provided in the publication. To extend our search strategy, the ‘Similar articles’ overview and references were checked to identify relevant articles which were missed with the initial search queries.

<u>Search query for pediatric pharmacokinetic studies:</u>

(Pharmacokinet*[Title/Abstract]) AND (***DEXAMETHASONE****[Title] OR ***DECADRON****[Title] OR ***ORADEXON****[Title]) AND (Infan*[Title] OR newborn*[Title] OR new-born*[Title] OR perinat*[Title] OR neonat*[Title] OR baby[Title] OR baby*[Title] OR babies[Title] OR prematur*[Title] OR preterm*[Title] OR toddler*[Title] OR minors[Title] OR minors*[Title] OR boy[Title] OR boys[Title] OR boyfriend[Title] OR boyhood[Title] OR girl*[Title] OR kid[Title] OR kids[Title] OR child[Title] OR child*[Title] OR children*[Title] OR schoolchild*[Title] OR schoolchild[Title] OR school child[Title] OR school child*[Title] OR adolescen*[Title] OR juvenil*[Title] OR youth*[Title] OR teen*[Title] OR under*age*[Title] OR pubescen*[Title] OR pediatrics[MeSH] OR pediatric*[Title] OR paediatric*[Title] OR peadiatric*[Title])

→ 19 results (1st of May 2023)

2. Pharmacokinetic studies

Table S1. Selected pharmacokinetic studies for dexamethasone model verification

	Study	N	Dose	Admini-stration	Health status	Age range (years)	% females	Ref
**1.1 Pediatric single dose***								
A	Nijstad et al. 2021	2	3 mg/m^2 with 3 mg/kg or 125 mg aprepitant	IV bolus<br>PO	Children with cancer undergoing chemotherapy	8.9-16.4	50 %	(1)
B	Nijstad et al. 2022†	18<br>47	<0.6 m^2 BSA: 4 mg q12h<br>6 mg/m^2 q6h<br><41.6 kg: 3 mg/m^2 q6h w/ 3 mg/kg apr<br>>41.6 kg: 3 mg/m^2 q6h w/ 125 mg apr	IV bolus<br><br>IV bolus, PO	Patients with a new oncological diagnosis planned to receive chemotherapy, such as medulloblastoma or osteosarcoma	0.6-16.5<br>0.7-17.9	44 %<br>40 %	(2)
C	Richter et al. 1983	12	0.3 mg/kg	IV bolus	Subglottic laryngitis	0.33-16	NS	(3)
D	Vallance et al. 2011	24<br>33<br>43	2.67 mg/m^2 q8h<br>4 mg/m^2 q8h<br>3 mg/m^2 q12h	All PO	Children past week 50 and prior to week 72 of the continuous chemotherapy phase of their treatment for ALL	5.0-18.1	38 %	(4)
**1.2 Pediatric multi dose**								
A	Jackson et al. 2019	92<br>82	5 mg/m^2 q12h for 14 days<br>3 mg/m^2 q12h for 28 days	PO<br>PO	At the first diagnosis of childhood ALL	1.3-18.7<br>1.5-17	49 %<br>45 %	(5)
B	Yang et al. 2008	165<br>49	2.67 mg/m^2 q8h for 8 days	PO	Children with ALL	1.0-9.9<br>10.0-18.8	46 %	(6)

Abbreviations: Acute lymphoblastic leukaemia (ALL), Body Surface Area (BSA), every 12 hours (q12h), every 8 hours (q8h), intravenous (IV), not specified (NS), number (N), oral (PO), reference (Ref), with (w/).*All studies were multi dose studies, yet PK samples were only taken after the first dose of dexamethasone. In the study of Nijstad et al. 2022, 80% of the samples were taken after the first dose. †Used multiple dosing strategies for dexamethasone in the study, the high emetogenicity doses are simulated as an bolus as 95% of the subjects received IV dexamethasone.

3. Model input parameters

Table S2. Drug-dependent input parameters for dexamethasone

	Parameter	Value
Physicochemical properties	Molecular weight (g/mol)	392.47
	Neutral species octanol : buffer partition coefficient (Log $P_{O:W}$)	1.84
	Compound type	Neutral
	Blood-to-plasma partition ratio (B/P)	0.93
	Fraction unbound (fu)	0.24
	Plasma binding protein	Human serum albumin (HSA)
Absorption	Absorption model	First-order
	Fraction available from dosage form (fa)	1 (10% CV; Coefficient of Variation)
	First order absorption rate constant (ka; 1/h)	0.8
	Lag time (h)	0.4
	Unbound fraction of drug in enterocytes (fu_{gut})	0.24
	A nominal flow in gut model (Q_{gut}; L/h)	13.463
	Human jejunum effective permeability ($P_{eff,man;}$ 10^{-4}cm/s)	3.61975
	Permeability Assay; values	Caco-2 (10^{-6} cm/s), 6.5:7.4, Passive & active - 23.4<br>Reference – Multiple; Maximum Extrapolated<br>$P_{eff,man}$ (10^{-4} cm/s) - 12
Distribution	Distribution model	Full
	Volume of distribution at steady state (V_{SS}; L/kg)	1.034 (Predicted)
	Prediction method	Method 2
	Kp Scalar	1.4 (Skin = 0.2)
Elimination	CYP3A4 *in vitro* intrinsic clearance (CL_{int})	0.15 (Pathway 1)
	Additional Liver CL (human liver microsomes) (µL/min/mg protein)	2.28
	Typical renal clearance for a 20-30 year healthy male (CL_R) (L/h)	0.39

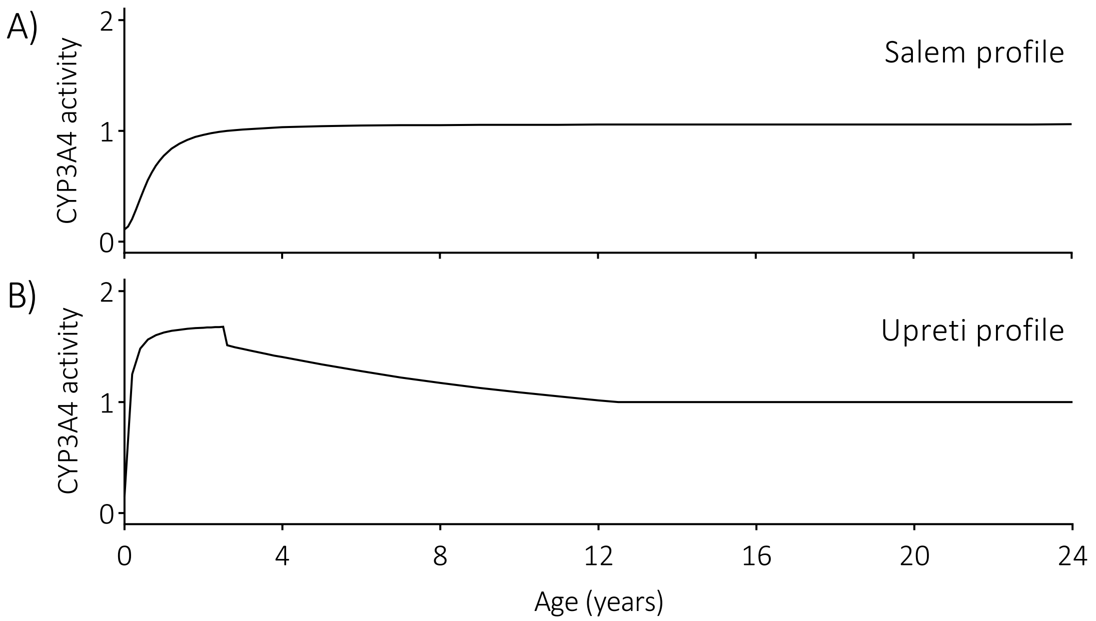

Figure S1. CYP3A4 ontogeny profiles. Change of hepatic CYP3A4 activity over age (adult relative activity is 1), according to A) the Salem equation: CYP3A4 activity = 0.11 + (((1.06 - 0.11)*$Age^{1.91}$) / ($0.64^{1.91}$ + $Age^{1.91}$)), and according to B) the Upreti equations: CYP3A4 activity below 2.5 years = 0.15 + (((1.7 - 0.15)*$Age^{1.3}$) / ($0.1^{1.3}$ + $Age^{1.3}$)) and CYP3A4 activity above 2.5 years = 0.7 + 1*$exp^{-0.01*(Age-0.5)}$.

4. Model verification

4.1 Model verification workflow and model performance assessment

First, the dexamethasone PBPK model was verified in the adult (non-pregnant) population which was performed in an earlier study (7). In short, simulations upon IV, PO and IM administration (single and multi dose) were compared with published PK data. Model performance was deemed acceptable to continue to a pregnant population, which is likewise acceptable to continue to a pediatric population.

Here, simulations with pediatric subjects receiving dexamethasone via IV or PO administration were conducted and evaluated with clinical data (Table S1). All studies with pediatric subjects that provide dexamethasone PK data, either plasma concentration data or PK parameters or both, were included in this study. CYP3A4 is the main metabolizing enzyme of dexamethasone (Table S2), yet there are two ontogeny profiles available in the Simcyp software (Figure S1). One is based on data from Salem et al. (8) and the other is based on data from Upreti & Wahlstrom (9). As no consensus has been reached on which profile to apply, all simulations for verification were conducted with both profiles to assess which profile results in more accurate predictions of dexamethasone PK in children. During the entire study period of the virtual trial, anatomical and physiological parameters of the pediaric population were adjusted according to age (function 'redefine subjects over time'). This function was, however, not compatible with a 'fixed trial design'. The virtual study designs were as follow:

- In the study of Nijstad et al. 2021 (1), aprepitant (i.e., a CYP3A4 inhibitor) was orally administered as 3 mg/kg (children <41.67 kg) or as a total dose of 125 mg (children ≥41.67 kg), hence two simulations were conducted with 2x51 and 2x49 subjects (fixed trial design), respectively, and simulation results were combined (i.e., a total of n=200 virtual subjects).
- In the study of Nijstad et al. 2022 (2), observed plasma concentrations were not linked to a specific dose. According to the dose regimens provided in the article, aprepitant was only co-administered with dexamethasone if dexamethasone was administered as 3 mg/m^2 every 6 hours (q6h) (corresponding to the previous simulation, children below and above 41.67 kg were simulated separately, i.e., fixed trial design). Further, one set of dexamethasone plasma values without aprepitant were compared with two separate simulations, namely the 4 mg q12h and 6 mg/m^2 q6h dexamethasone administration, as it was not possible to allocate the observed values to a specific dosing regimen. The 2 mg q6h dose regimen was not included as only few simulated individuals had a body surface area <0.6.

- In the study of Richter et al. (3), a straight forward single dose simulation of 0.3 mg/kg was conducted with 10 trials and each 10 subjects (i.e., 10x10).
- In the study of Vallance et al. (4), children were allocated to one of the three groups each receiving different doses. 48 virtual subjects received 2.67 mg/m^2 q8h (2x24), 66 received 4 mg/m^2 q8h (2x33) and 86 received 3 mg/m^2 q12h (2x43) dexamethasone. The number of children receiving each dosing strategy was obtained from another article (10). Predicted PK parameters were derived from all 200 virtual subjects.
- In the study of Jackson et al. (5), the standard (i.e., 3 mg/m^2 q12h for 28 days) and short (i.e., 5 mg/m^2 q12h for 14 days) dosing strategies were separately simulated (10x10).
- In the study of Yang et al. (6), three separate simulations were conducted to obtain predicted PK values, i.e., for children below 9.92 years, above 10 years and all ages together receiving 2.67 mg/m^2 q8h (10x10).

Predictive performance of PBPK models was evaluated by 1) calculating the ratio of predicted-to-observed PK parameters, and by 2) a visual predictive check (VPC) of the agreement of the predicted and observed plasma concentration-time profile. Ratios within 0.5 to 2-fold range were considered acceptable. For a visual check, observed plasma concentration-time profiles were extracted from literature, digitalized with WebPlotDigitizer v4.6, and compared to predicted plasma concentration-time curves. To give an indication of overall model performance, prediction errors (PE) were calculated as described previously (11). Additionally, for the studies Jackson et al. and Yang et al., individual clearance values were as well extracted with WebPlotDigitizer and visually compared with predicted individual clearance values.

4.2 Model verification results

Predicted-to-observed PK parameter ratios are shown in Figure S2. For dexamethasone predictions with the Salem profile, 86.67% of predicted-to-observed PK ratios were within 2-fold, 66.67% within 1.5-fold and 46.67% within 1.25-fold range, while for predictions with the Upreti profile 86.67% of PK ratios were within 2-fold range, 70% within 1.5-fold, and 43.33% within 1.25-fold range.

VPCs for dexamethasone predictions in pediatrics with the Salem profile are shown in Figure S3 while Figure S4 shows the predictions with the Upreti profile. Corresponding prediction errors for all simulations are shown in Figure S5 and S6. A high interindividual variability in the observed and predicted apparent oral clearance is observed (Both Figure S3 and S4I,J&L). Furthermore, oral exposure is somewhat underpredicted (Figure S3 and

S4G&H). Though, predictions of dexamethasone concentrations upon IV administration are very accurate (Figure S3 and S4A&C-E).

A minimal difference between predictions with the Salem and Upreti profile are observed. Still, predicted clearance is more in line with observed clearances by Nijstad et al., Vallance et al., and Yang et al, when the Upreti ontogeny profile is applied. However, only the apparent oral clearance corresponding to the Jackson et al. study are predicted worse with the Uperti profile, yet the observed clearance is an estimated value derived from a population-PK analysis. Hence, the Upreti profile is considered to be the appropriate maturation profile to predict dexamethasone PK in children and this model was therefore used for the actual dosing simulations.

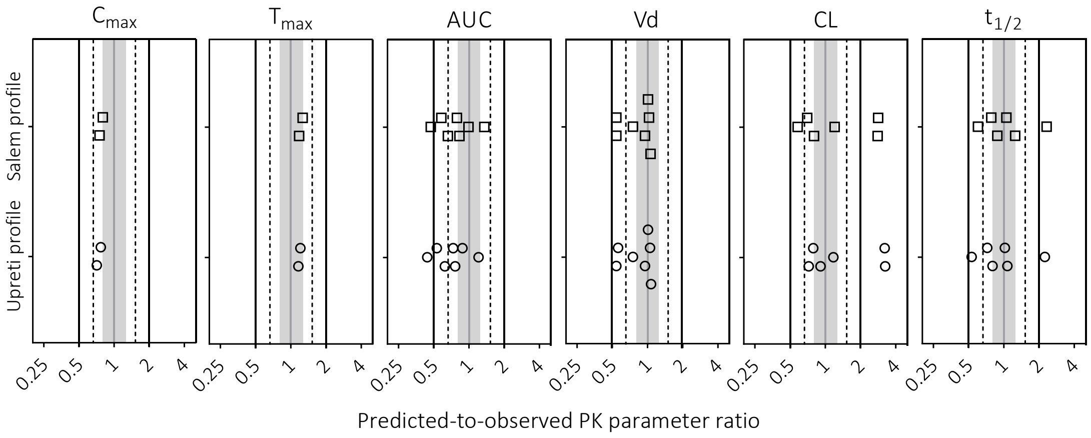

Figure S2. Predicted-to-observed ratios of the maximum concentration (C_{max}), time to peak concentration (T_{max}), area under the curve (AUC), volume of distribution (Vd), clearance (CL), and half-life ($t_{1/2}$) for dexamethasone in pediatrics. Single symbols represent a predicted-to-observed ratio of a single pharmacokinetic study with Salem or Upreti profile (Figure S1). The black lines represent the 2-fold range, the dashed lines the 1.5-fold range, the gray shaded area represent the 1.25-fold range and the gray line represents the unity line.

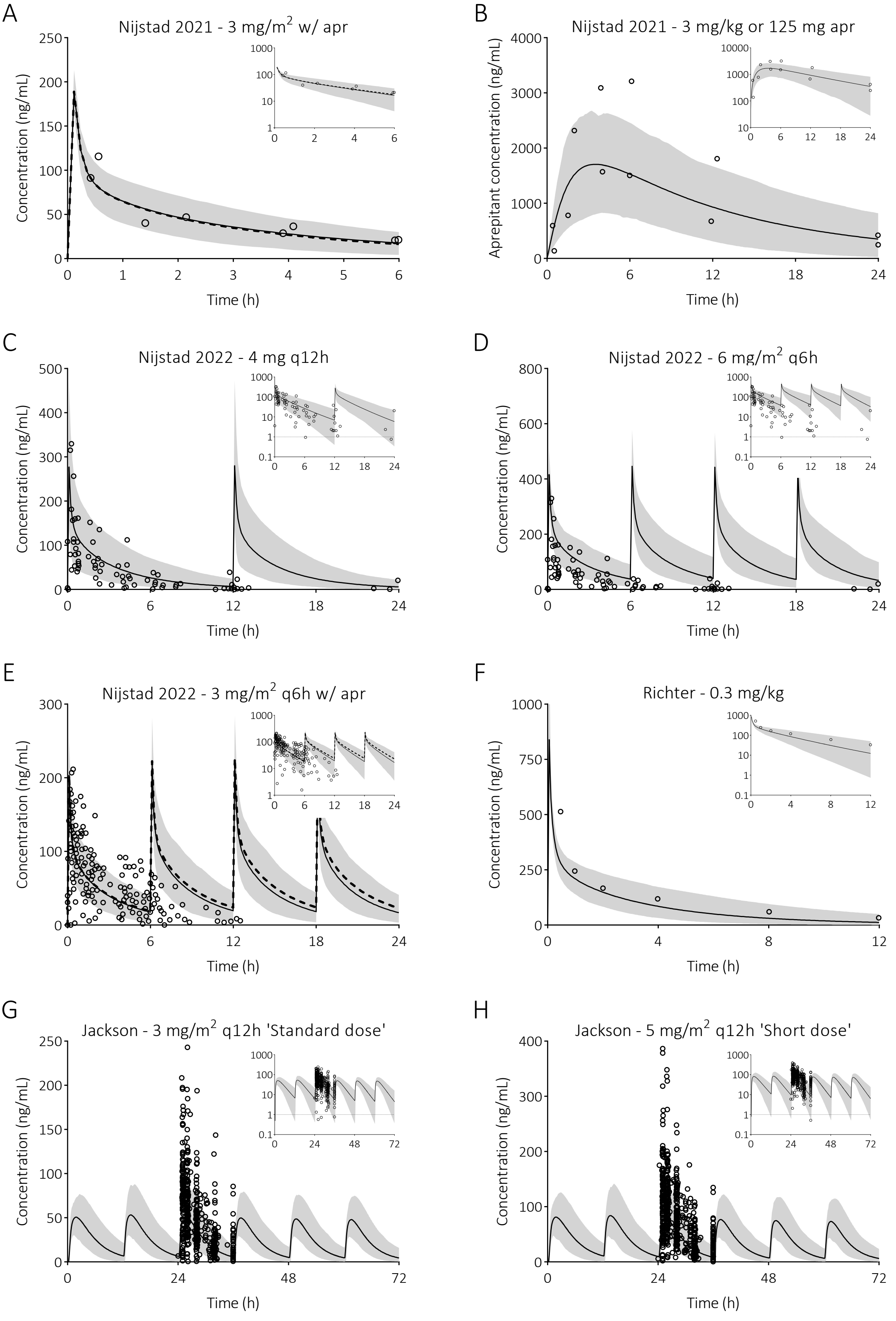
A
Nijstad 2021 - 3 mg/m2 w/ apr
Concentration (ng/mL)
Time (h)
B
Nijstad 2021 - 3 mg/kg or 125 mg apr
Aprepitant concentration (ng/mL)
Time (h)
C
Nijstad 2022 - 4 mg q12h
Concentration (ng/mL)
Time (h)
D
Nijstad 2022 - 6 mg/m2 q6h
Concentration (ng/mL)
Time (h)
E
Nijstad 2022 - 3 mg/m2 q6h w/ apr
Concentration (ng/mL)
Time (h)
F
Richter - 0.3 mg/kg
Concentration (ng/mL)
Time (h)
G
Jackson - 3 mg/m2 q12h 'Standard dose'
Concentration (ng/mL)
Time (h)
H
Jackson - 5 mg/m2 q12h 'Short dose'
Concentration (ng/mL)
Time (h)

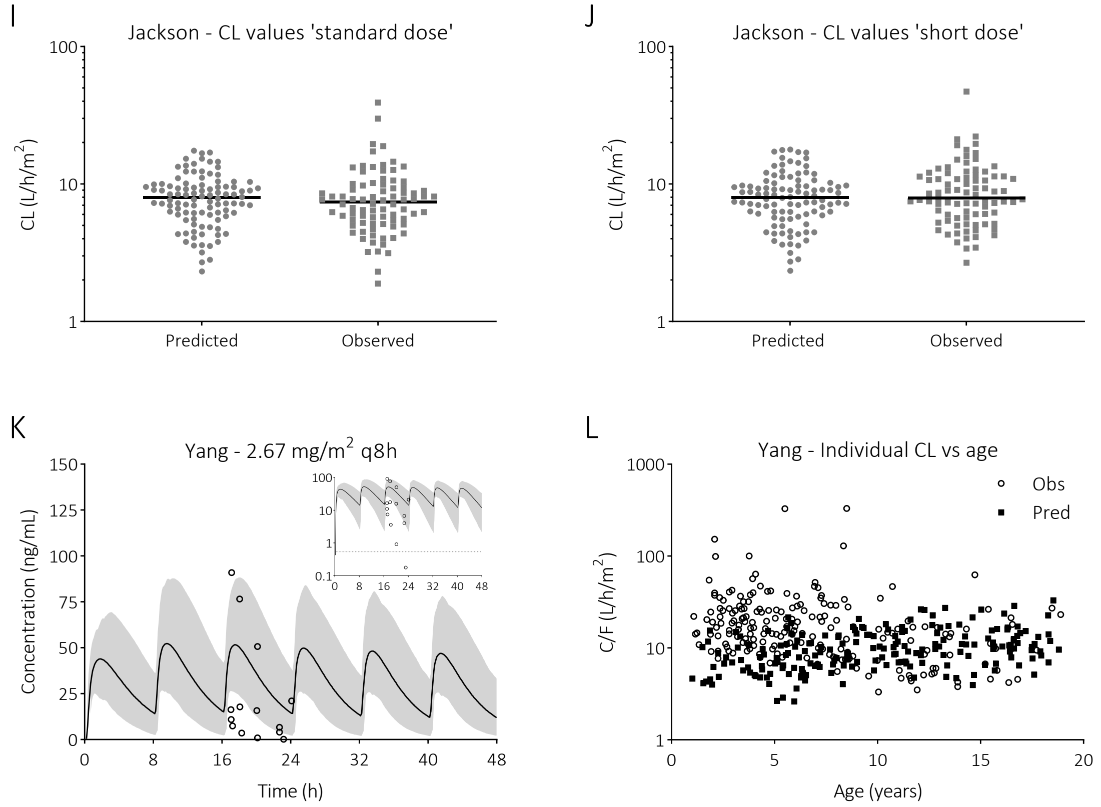

Figure S3. Prediction of dexamethasone plasma concentration-time profiles in pediatric individuals with the Salem CYP3A4 profile. The solid line is the predicted mean of the simulated population and the shaded area represents the 5th to 95th percentile of the virtual population. The dashed line in A) and E) indicate the predicted mean with apreptitant. Open circles are the observed data: A & B (1), C-E (2), F (3), G-J (5), K and L (6). B) shows observed and predicted aprepitant values to indicate accurate prediction of the inhibitor drug. I) and J) show individual observed and predicted CL values with the median CL for the separate dosing strategies. L) shows observed and predicted individual CL values over the studied age range. The dashed horizontal lines in the insets represent the reported lower limit of quantification (LLOQ) of the clinical studies. Provided prediction error (PE) is the median of the PEs per observation. Abbreviations: clearance (CL), every 12 hours (q12h), every 6 hours (q6h), every 8 hours (q8h), observed (Obs), predicted (Pred).

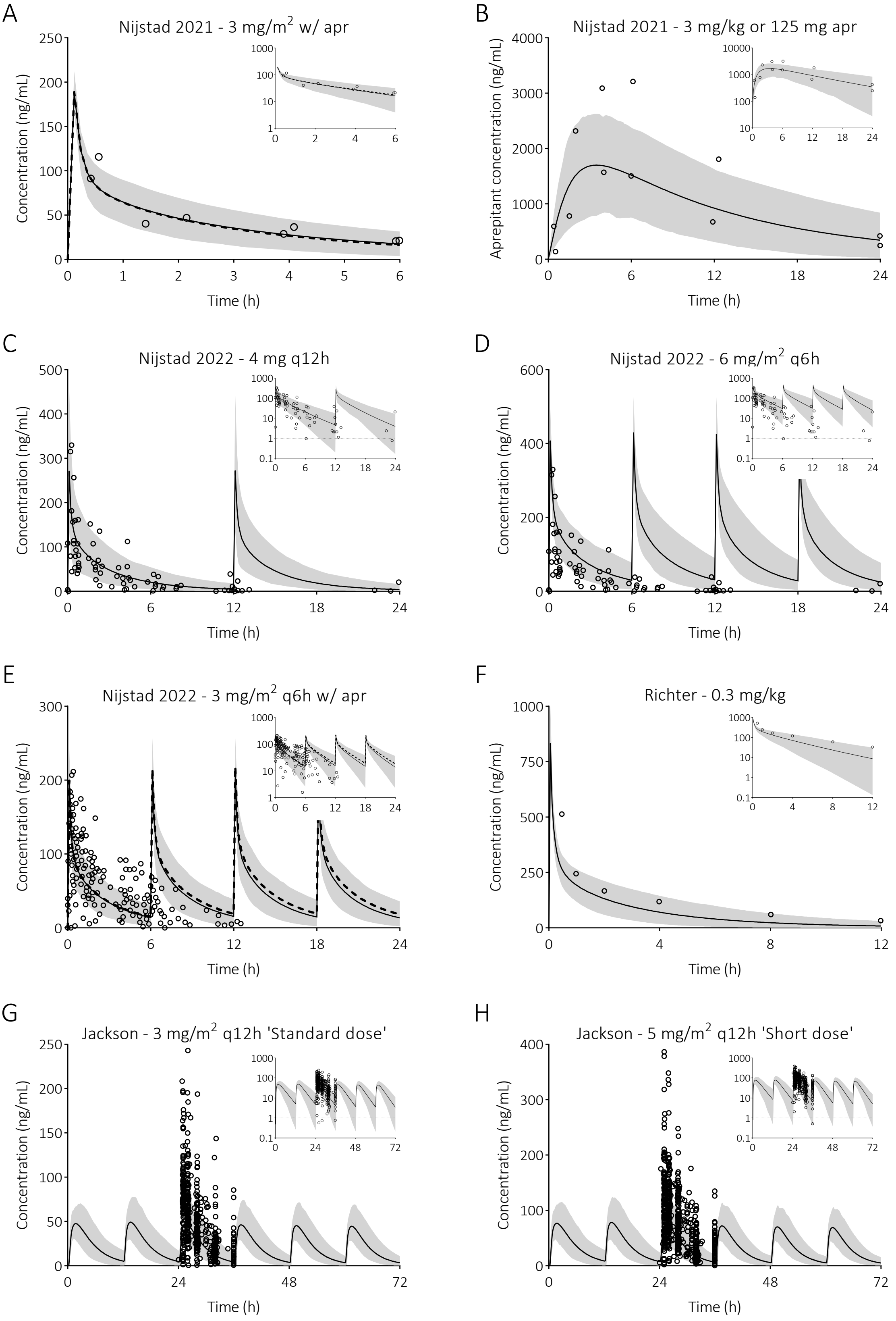
A
Nijstad 2021 - 3 mg/m2 w/ apr
Concentration (ng/mL)
Time (h)
B
Nijstad 2021 - 3 mg/kg or 125 mg apr
Aprepitant concentration (ng/mL)
Time (h)
C
Nijstad 2022 - 4 mg q12h
Concentration (ng/mL)
Time (h)
D
Nijstad 2022 - 6 mg/m2 q6h
Concentration (ng/mL)
Time (h)
E
Nijstad 2022 - 3 mg/m2 q6h w/ apr
Concentration (ng/mL)
Time (h)
F
Richter - 0.3 mg/kg
Concentration (ng/mL)
Time (h)
G
Jackson - 3 mg/m2 q12h 'Standard dose'
Concentration (ng/mL)
Time (h)
H
Jackson - 5 mg/m2 q12h 'Short dose'
Concentration (ng/mL)
Time (h)

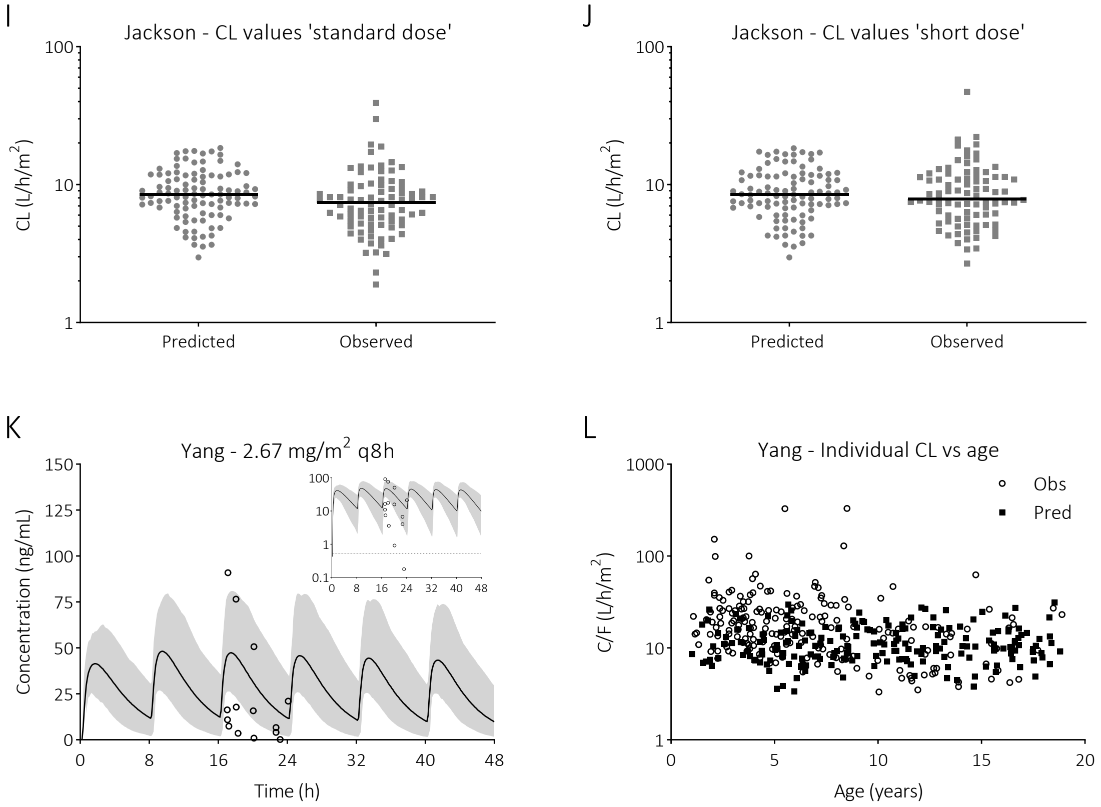

Figure S4. Prediction of dexamethasone plasma concentration-time profiles in pediatric individuals with the Upreti CYP3A4 profile. The solid line is the predicted mean of the simulated population and the shaded area represents the 5th to 95th percentile of the virtual population. The dashed line in A) and E) indicate the predicted mean with aprepritant. Open circles are the observed data: A & B (1), C-E (2), F (3), G-J (5), K and L (6). B shows observed and predicted aprepitant values to indicate accurate prediction of the inhibitor drug. I and J show individual observed and predicted CL values with the median CL for the separate dosing strategies. L shows observed and predicted individual CL values over the studied age range. The dashed horizontal lines in the insets represent the reported lower limit of quantification (LLOQ) of the clinical studies. Provided prediction error (PE) is the median of the PEs per observation. Abbreviations: clearance (CL), every 12 hours (q12h), every 6 hours (q6h), every 8 hours (q8h), observed (Obs), predicted (Pred).

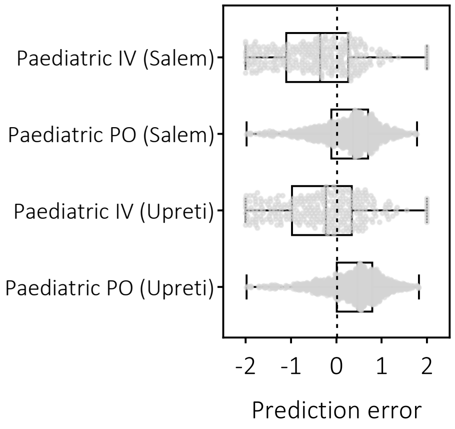

Figure S5. Box-whisker plots for the prediction errors (PE) describing observed vs predicted plasma concentrations. Data are shown per dosing route for pediatric observations per CYP3A4 ontogeny profile, i.e., Salem or Upreti profile (Figure S1). PE values for all observations (i.e., all studies and all time points), irrespective of time, are presented as gray in the whisker plots. The box-whiskers indicate the quartiles with the median and the minimum and maximum PE value. Note that positive values indicate an underprediction and negative values an overprediction by the model. Full plots of PE values over time are shown in Figure S6.

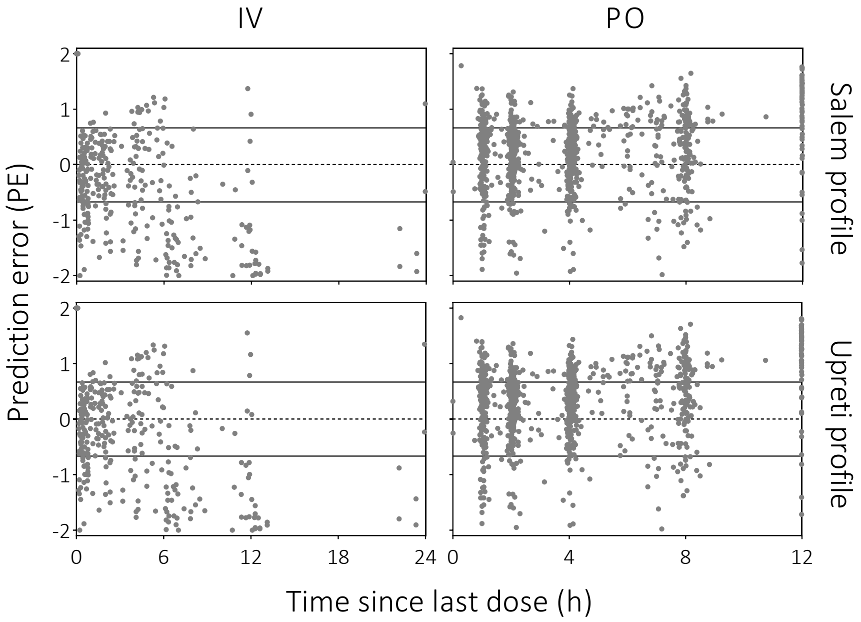

Figure S6. Prediction errors (PE) over time since last dose for pediatric observations. Intravenous (IV) and oral (PO) studies presented separately with Salem or Upreti profile (Figure S1). Gray dots are PE values for each observation.

4.3 Discussion applied CYP3A4 ontogeny profile

CYP3A4 is the main metabolizing enzyme of dexamethasone, hence having a great impact on drug clearance. Determining maturation profiles of metabolizing enzymes in pediatrics can be done via multiple approaches (12). Although requiring much effort, a varying set of *in vitro*- and *in vivo*-based CYP3A4 ontogeny functions are available in literature (8, 9, 13, 14). While some ontogeny functions are comparable, others are contradicting. Still, no consensus has been reached on which function reflects the clinical situation best. We evaluated both the Salem and Upreti ontogeny profiles, which are contradicting. The function proposed by Upreti suggests a rapid increase in activity upon birth exceeding adult levels in the age range of 0.1 and 11 years while Salem suggests a more graduate increase of activity reaching adult levels by 2.5 years (Figure S1). We determined that using the profile of Upreti et al, dexamethasone PK is predicted more accurately in pediatric subjects. This is in line with the observations of Johnson et al. who compared the use of both profiles to predict pediatric midazolam PK (15). Comparing the profiles, the difference in model performance was more striking compared to our results. For dexamethasone, only pediatric PK data in large age groups are available (e.g., 1 month – 18 years) while for midazolam PK data in younger and more specific age groups are available. Here improved predictions were particularly seen in the approximate age range 0-1 year and to a lesser extent in the ~2-4 year range. Dexamethasone PK data in these specific age groups are lacking. The study of Yang et al. (6) resembles most accurately this age range (i.e., ~1-10 years) where the CL and $t_{1/2}$ ratio improves from 0.58 and 1.25 with the Salem profile to 0.79 and 1.02 with the Upreti profile, respectively. Furthermore, other PBPK studies have evaluated both ontogeny profiles, but then for the drugs tacrolimus (16), mefloquine (17), imatinib (18) and ivabradine (19). Here, predictions of mefloquine PK are more accurate with the Upreti profile (0.5-2 years age range), predictions of imatinib result in similar performances (>2 years of age), whereas tacrolimus PK was better predicted with the Salem profile which adjusts for disease effect (1-16 year old children).

5. Simulating alternative dosing regimens

Model application approach – Exploring alternative dosing scenarios

After model verification, the model was applied to evaluate various alternative dosing scenarios upon IV administration in children. First, the current Dutch dosing regimen to prevent post-extubation stridor was simulated (Table S3) (20). The pediatric age range was divided in smaller subgroups for our simulations, i.e., 12-18 years, 6-12 years, 2-6 years, 1-2 years, 6-12 months, 3-6 months, 1-3 months, 2-4 weeks, and 0-2 weeks. Several dosing schedules were simulated that have shown to be effective in children to prevent post-extubation stridor, described as a 'high late' (i.e., 3 times 0.5 mg/kg IV doses q6h) and 'low early' (i.e., 6 times 0.25 mg/kg IV doses q6h) (21). As well as the doubled recommended dose for subglottic laryngitis, double administration of 0.15 mg/kg IV (22). It was decided to match total exposure (AUC) of the different pediatric age ranges to the exposure of the 2-6 years age group. No exposure data is available in adults and most evidence of effective doses was available for the 2-6 years age range. Additionally, highest peak exposures (C_{max}) during the treatment duration (i.e., 0-48 hours) were compared between age groups and dosing regimens.

Alternative dosing regimen results

The plasma concentration-time profiles for the 2-6 years age group of each dosing schedule (including the current Dutch Paediatric Formulary (DPF) recommendations) are provided in Figure S7. Simulated total and peak exposures of the current dosing regimens of dexamethasone per paediatric age group are shown in Figure S8. It is noticeable that total exposure in neonates are higher upon the current dosing recommendations for the other age groups (without the repeated dose), especially for the 0-2 weeks old age group. Median AUC_{0-48} are 2578 ng/mL*h (25th-75th percentile: 2001 - 3650) and 3949 ng/mL*h (25th-75th percentile: 2966 - 5665) for neonates 2-4 and 0-2 weeks postnatal age (PNA), respectively. The AUC_{0-48} of the 2-6 years age group is 1199 ng/mL*h (25th-75th percentile: 975 - 1616). Whereas peak exposures are considerably lower in the neonatal age groups compared to the older children (≥1 month PNA), namely: 1199 ng/mL (25th-75th percentile: 1158 - 1267) in children 0-2 weeks 1173 ng/mL (25th-75th percentile: 1132 - 1226) in children 2-4 weeks and 2302 ng/mL (25th-75th percentile: 2228 - 2396) in children 2-6 years.

The predicted total and peak exposures of the 'high late' and 'low early' dosing schedules from Parajuli et al. are shown in Figure S9 and of the subglottic laryngitis dosing schedule is depicted in Figure S10. The predicted median AUC_{0-48} with the 25th and 75th percentile range for the 'high late', 'low early' and subglottic laryngitis dosing schedule are

3174 ng/mL*h (2672 - 4339), 3082 ng/mL*h (2572 - 4146), and 710 ng/mL*h (583 - 960), respectively. The doses for all age groups are exposure matched to these predicted total exposures per dosing schedule. No dosing adjustments were required for the age groups 3-6 months, 6-12 months and 1-2 years (e.g., 0.5 mg/kg). Compared to the 2-6 years age group, independent of dosing schedule, the dose should be reduced with 20% for the age groups 1-3 months and 6-12 years (e.g., 0.4 mg/kg vs 0.5 mg/kg). For the age groups 2-4 weeks and 12-18 years, the dose should be reduced with 40% (e.g., 0.3 vs. 0.5 mg/kg) and with 60% reduced for the 0-2 weeks age group (e.g., 0.2 vs. 0.5 mg/kg). The final model-informed doses per age group and per dosing schedule are provided in Table S4.

Table S3. Current dosing regimens on the Dutch Paediatric Formulary for prophylaxis of post-extubation stridor (20).

Prophylaxis post-extubation stridor
**Intravenous**<br>○ **Premature neonates, gestational age <37 weeks**<br>▪ Loading dose: At least 4 hours prior to extubation: 0.25 mg/kg/dose, once.<br>▪ Maintenance dose: 0.25 mg/kg/dose 8 and 16 hours after the first dose (3 doses in total), i.e., a cumulative dose of 0.75 mg/kg.<br><br>○ **Term neonates**<br>▪ Loading dose: At least 4 hours prior to extubation: 0.25 mg/kg/dose, once.<br>▪ Maintenance dose: 0.25 mg/kg/dose 8 and 16 hours after the first dose (3 doses in total), i.e., a cumulative dose of 0.75 mg/kg.<br><br>○ **1 month to 18 years and <40 kg**<br>▪ Prior to extubation: 0.5 mg/kg/dose, repeat if necessary. Max: 40 mg/day. I.e., a cumulative dose of 1 mg/kg.<br><br>○ **1 month to 18 years and ≥40 kg**<br>▪ Prior to extubation: 20 mg/dose, repeat if necessary. Max: 40 mg/day. I.e., a cumulative dose of ≤1 mg/kg (depending on bodyweight)

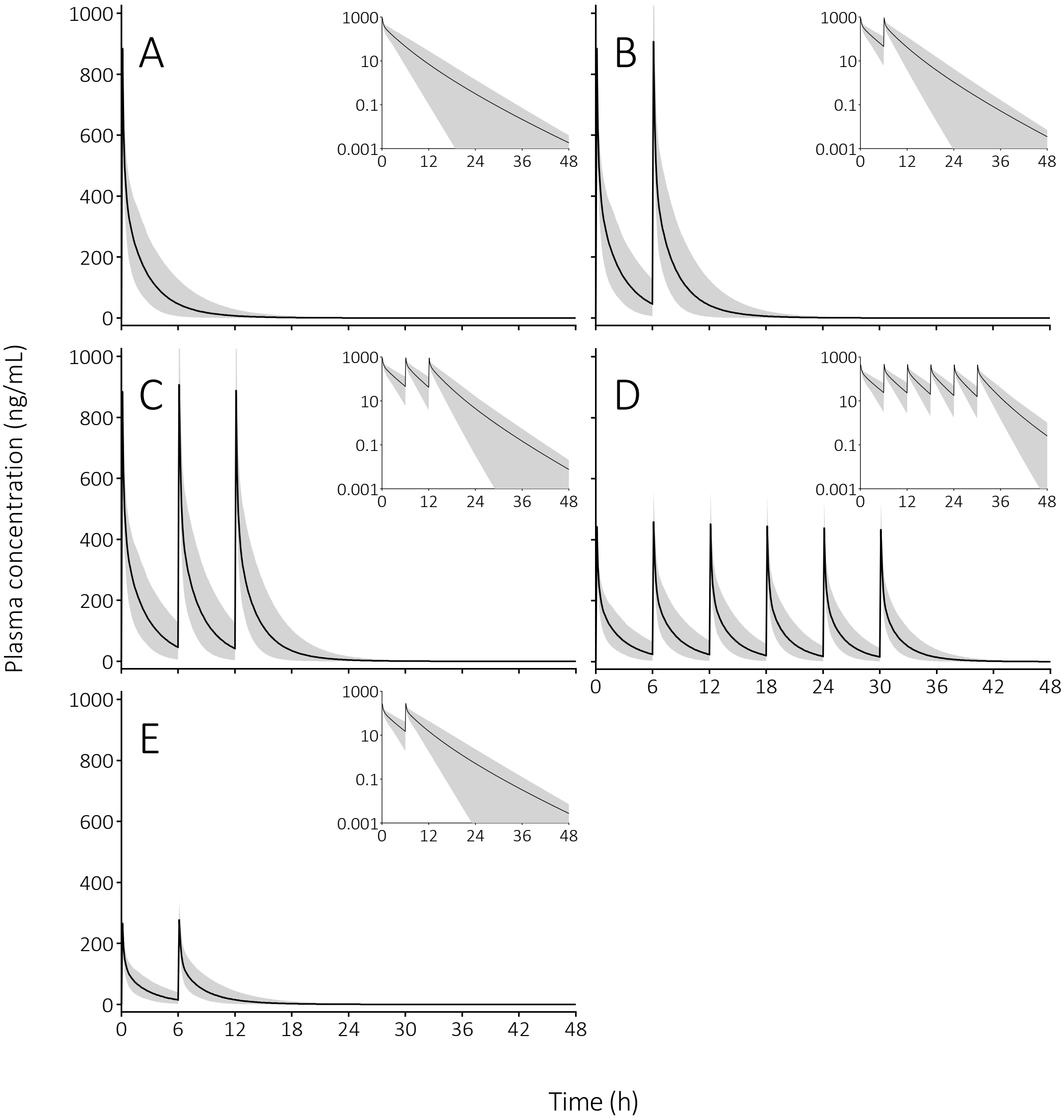

Figure S7. Plasma concentration-time profiles of alternative dosing recommendations of dexamethasone in pediatric individuals aged 2 to 6 years. A) Current Dutch Paediatric Formulary (DPF) dose (single dose), B) Current DPF with repeated dose, C) 'High late' dosing recommendation (21), D) 'Low early' dosing recommendation (21), E) Double subglottic laryngitis dose (22). The solid line is the predicted mean of the simulated population and the shaded area represents the 5th to 95th percentile of the virtual population.

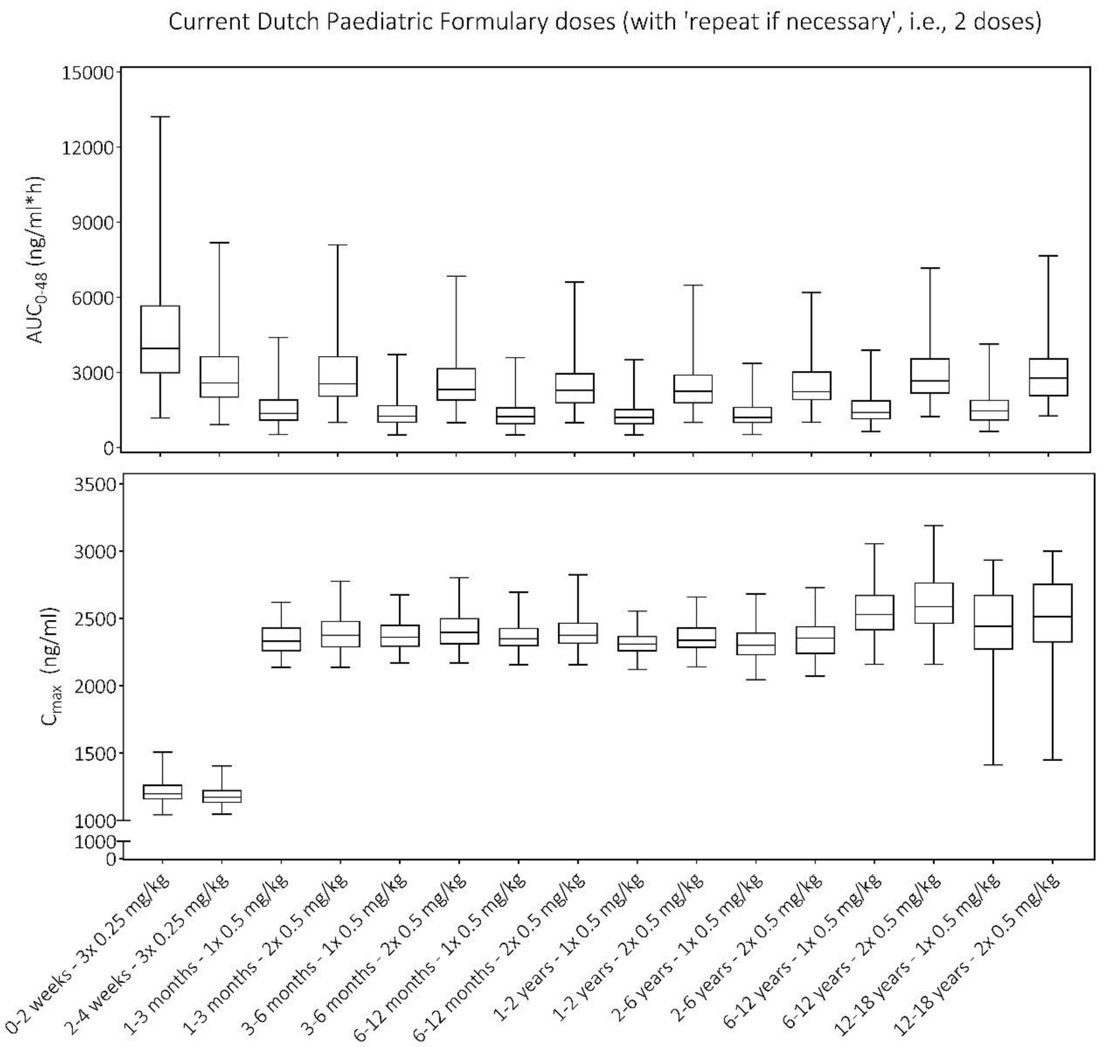

Figure S8. Prediction of dexamethasone total exposure over 48 hours (AUC_{0-48}) and highest peak exposure (C_{max}) during the treatment period following the current intravenous Dutch Paediatric Formulary doses per age group. Maximum dose per administration is 20 mg. The boxes indicate the median prediction with the 25th and 75th percentile ranges, while the whiskers indicate the predicted minimum and maximum values of the simulated populations.

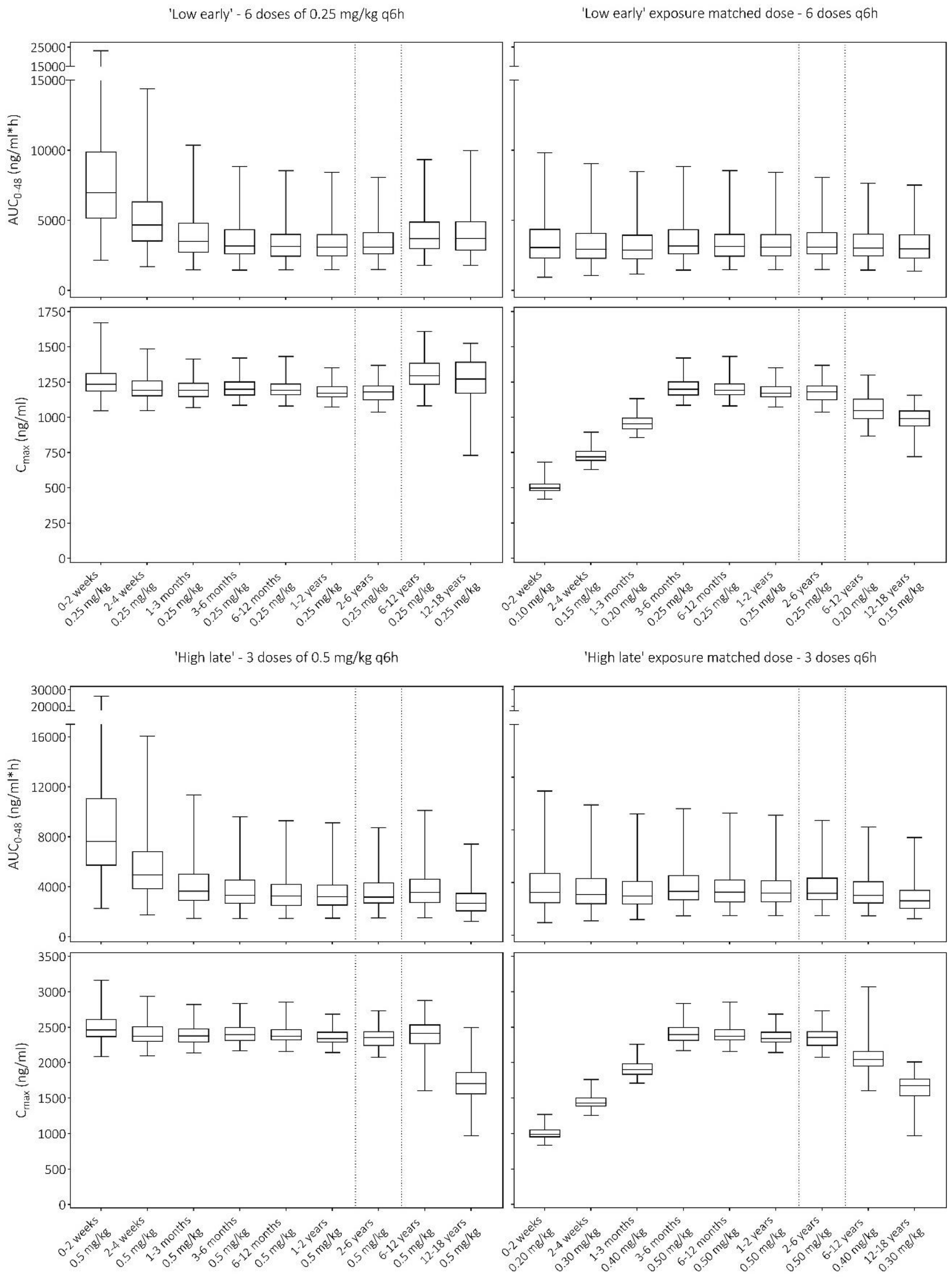

Figure S9. Prediction of dexamethasone total exposure over 48 hours (AUC_{0-48}) and highest peak exposure (C_{max}) during the treatment period following the 'Low early' (top part, left) and 'High late' (bottom part, left) dosing schedule of Parajuli et al. (21) with the corresponding exposure matched dose per age group (right column). Maximum dose per administration is 20 mg. The boxes indicate the median prediction with the 25th and 75th percentile ranges, while the whiskers indicate the predicted minimum and maximum values of the simulated populations. The age group 2-6 years is indicated between dashed lines.

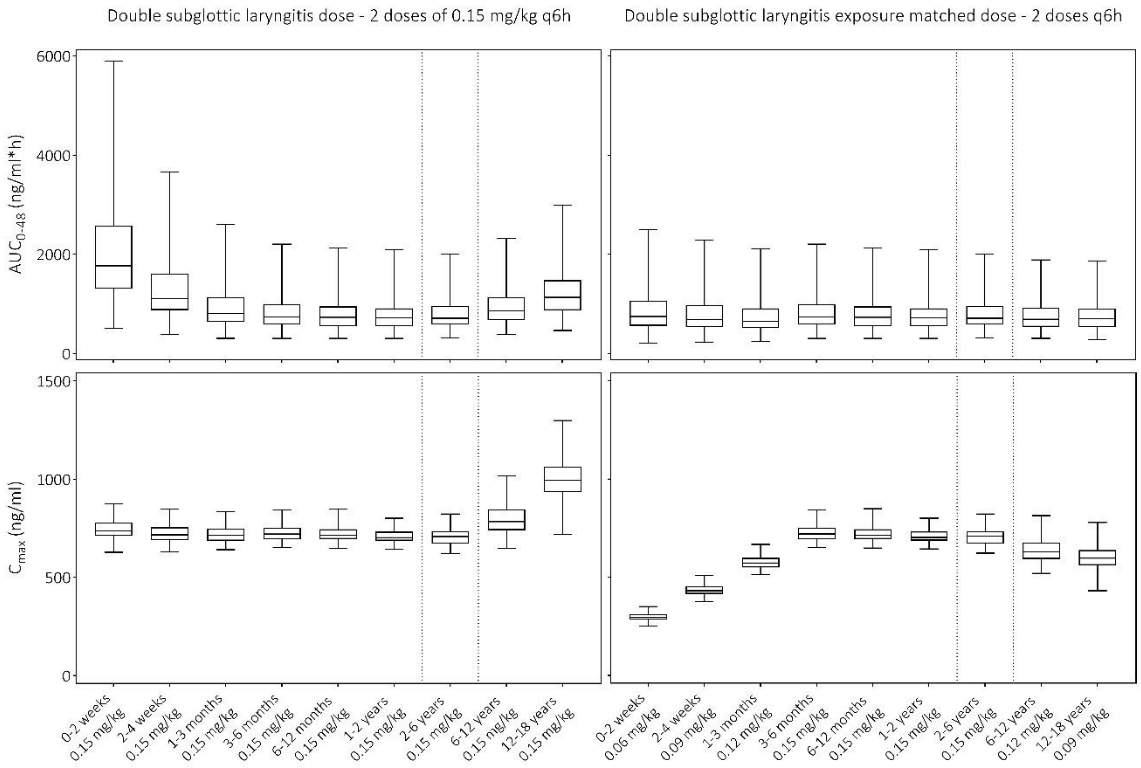

Figure S10. Prediction of dexamethasone total exposure over 48 hours (AUC_{0-48}; top row) and highest peak exposure (C_{max}; bottom row) during the treatment period following the double subglottic laryngitis dose (22) with the corresponding exposure matched dose per age group (right column). Maximum dose per administration is 20 mg. The boxes indicate the median prediction with the 25th and 75th percentile ranges, while the whiskers indicate the predicted minimum and maximum values of the simulated populations. The age group 2-6 years is indicated between dashed lines.

Table S4. Proposed model-informed dosing regimens for prophylaxis of post-extubation stridor.

Age group	'Low early' - Start 12-24h prior 6 doses q6h	'High late' - Start 6-12h prior 3 doses q6h	'Subglottic laryngitis' – Start 1-6h prior 2 doses q6h
12-18 years	0.15 mg/kg	0.30 mg/kg	0.09 mg/kg
6-12 years	0.20 mg/kg	0.40 mg/kg	0.12 mg/kg
3 months - 6 years	0.25 mg/kg	0.50 mg/kg	0.15 mg/kg
1-3 months	0.20 mg/kg	0.40 mg/kg	0.12 mg/kg
Term neonates (2-4 weeks)	0.15 mg/kg	0.30 mg/kg	0.09 mg/kg
Term neonates (0-2 weeks)	0.10 mg/kg	0.20 mg/kg	0.06 mg/kg

6. References

1. Nijstad AL, Tibben MM, Gebretensae A, Rosing H, de Vos-Kerkhof E, Zwaan CM, et al. Development and validation of a combined liquid chromatography tandem-mass spectrometry assay for the quantification of aprepitant and dexamethasone in human plasma to support pharmacokinetic studies in pediatric patients. J Chromatogr B Analyt Technol Biomed Life Sci. 2021;1171:122639.
2. Nijstad AL, de Vos-Kerkhof E, Enters-Weijnen CF, van de Wetering MD, Tissing WJE, Tibben MM, et al. Overestimation of the effect of (fos)aprepitant on intravenous dexamethasone pharmacokinetics requires adaptation of the guidelines for children with chemotherapy-induced nausea and vomiting. Support Care Cancer. 2022;30(12):9991-9.
3. Richter O, Ern B, Reinhardt D, Becker B. Pharmacokinetics of dexamethasone in children. Pediatr Pharmacol (New York). 1983;3(3-4):329-37.
4. Vallance K, Liu W, Mandrell BN, Panetta JC, Gattuso JS, Hockenberry M, et al. Mechanisms of dexamethasone-induced disturbed sleep and fatigue in paediatric patients receiving treatment for ALL. Eur J Cancer. 2010;46(10):1848-55.
5. Jackson RK, Liebich M, Berry P, Errington J, Liu J, Parker C, et al. Impact of dose and duration of therapy on dexamethasone pharmacokinetics in childhood acute lymphoblastic leukaemia-a report from the UKALL 2011 trial. Eur J Cancer. 2019;120:75-85.
6. Yang L, Panetta JC, Cai X, Yang W, Pei D, Cheng C, et al. Asparaginase may influence dexamethasone pharmacokinetics in acute lymphoblastic leukemia. J Clin Oncol. 2008;26(12):1932-9.

7. Van Der Heijden JEM, Van Hove H, Van Elst NM, Van Den Broek P, Van Drongelen J, Scheepers HCJ, et al. Optimization of the betamethasone and dexamethasone dosing regimen during pregnancy: a combined placenta perfusion and pregnancy physiologically based pharmacokinetic modeling approach. Am J Obstet Gynecol. 2024.
8. Salem F, Johnson TN, Abduljalil K, Tucker GT, Rostami-Hodjegan A. A re-evaluation and validation of ontogeny functions for cytochrome P450 1A2 and 3A4 based on in vivo data. Clin Pharmacokinet. 2014;53(7):625-36.
9. Upreti VV, Wahlstrom JL. Meta-analysis of hepatic cytochrome P450 ontogeny to underwrite the prediction of pediatric pharmacokinetics using physiologically based pharmacokinetic modeling. J Clin Pharmacol. 2016;56(3):266-83.
10. Hinds PS, Hockenberry MJ, Gattuso JS, Srivastava DK, Tong X, Jones H, et al. Dexamethasone alters sleep and fatigue in pediatric patients with acute lymphoblastic leukemia. Cancer. 2007;110(10):2321-30.
11. van der Heijden JEM, Freriksen JJM, de Hoop-Sommen MA, van Bussel LPM, Driessen SHP, Orlebeke AEM, et al. Feasibility of a Pragmatic PBPK Modeling Approach: Towards Model-Informed Dosing in Pediatric Clinical Care. Clin Pharmacokinet. 2022;61(12):1705-17.
12. van Groen BD, Allegaert K, Tibboel D, de Wildt SN. Innovative approaches and recent advances in the study of ontogeny of drug metabolism and transport. Br J Clin Pharmacol. 2022;88(10):4285-96.
13. Johnson TN, Rostami-Hodjegan A, Tucker GT. Prediction of the clearance of eleven drugs and associated variability in neonates, infants and children. Clin Pharmacokinet. 2006;45(9):931-56.
14. Edginton AN, Schmitt W, Voith B, Willmann S. A mechanistic approach for the scaling of clearance in children. Clin Pharmacokinet. 2006;45(7):683-704.
15. Johnson TN, Howgate EM, de Wildt SN, Turner MA, Rowland Yeo K. Use of Developmental Midazolam and 1-Hydroxymidazolam Data with Pediatric Physiologically Based Modeling to Assess Cytochrome P450 3A4 and Uridine Diphosphate Glucuronosyl Transferase 2B4 Ontogeny In Vivo. Drug Metab Dispos. 2023;51(8):1035-45.
16. Emoto C, Johnson TN, Hahn D, Christians U, Alloway RR, Vinks AA, Fukuda T. A Theoretical Physiologically-Based Pharmacokinetic Approach to Ascertain Covariates Explaining the Large Interpatient Variability in Tacrolimus Disposition. CPT Pharmacometrics Syst Pharmacol. 2019;8(5):273-84.
17. Johnson TN, Cleary Y, Parrott N, Reigner B, Smith JR, Toovey S. Development of a physiologically based pharmacokinetic model for mefloquine and its application alongside a clinical effectiveness model to select an optimal dose for prevention of malaria in young Caucasian children. Br J Clin Pharmacol. 2019;85(1):100-13.
18. Adiwidjaja J, Boddy AV, McLachlan AJ. Implementation of a Physiologically Based Pharmacokinetic Modeling Approach to Guide Optimal Dosing Regimens for Imatinib and Potential Drug Interactions in Paediatrics. Front Pharmacol. 2019;10:1672.

19. Lang J, Vincent L, Chenel M, Ogungbenro K, Galetin A. Impact of Hepatic CYP3A4 Ontogeny Functions on Drug-Drug Interaction Risk in Pediatric Physiologically-Based Pharmacokinetic/Pharmacodynamic Modeling: Critical Literature Review and Ivabradine Case Study. Clin Pharmacol Ther. 2021;109(6):1618-30.
20. Kinderformularium. Kinderformularium - Dexamethason [updated 12 July 2019] Date cited: 10 May 2023. Available from: https://www.kinderformularium.nl/geneesmiddel/182/dexamethason.
21. Parajuli B, Baranwal AK, Kumar MP, Jayashree M, Takia L. Twenty-four-hour pretreatment with low dose (0.25 mg/kg/dose) versus high dose (0.5 mg/kg/dose) dexamethasone in reducing the risk of postextubation airway obstruction in children: A randomized open-label noninferiority trial. Pediatr Pulmonol. 2021;56(7):2292-301.
22. Nederlandse Vereniging voor Kindergeneeskunde - Dexamethason bij Laringitis Subglottica [updated 28 July 2014] Date cited: 1 August 2023. Available from: http://www.nvk.nl/.
